# Supplementary material for: GSK3β Inhibition Promotes Efficient Myeloid and Lymphoid Hematopoiesis from Non-human Primate-Induced Pluripotent Stem Cells
Source: Stem Cell Reports. 2016 Jan 21;6(2):243–56. doi: 10.1016/j.stemcr.2015.12.010 (PMC4750098; doi:10.1016/j.stemcr.2015.12.010)
Supplement: Document S1. Supplemental Experimental Procedures, Figures S1–S5, and Tables S1 and S2 [file mmc1.pdf]

Stem Cell Reports, Volume *6*

Supplemental Information

GSK3β Inhibition Promotes Efficient Myeloid and Lymphoid Hematopoiesis from Non-human Primate-Induced Pluripotent Stem Cells

Saritha S. D'Souza, John Maufort, Akhilesh Kumar, Jiuchun Zhang, Kimberley Smuga-Otto, James A. Thomson, and Igor I. Slukvin

Supplementary Information

GSK3β Inhibition Promotes Efficient Myeloid and Lymphoid Hematopoiesis from Nonhuman Primate Induced Pluripotent Stem Cells

Saritha S. D'Souza, John Maufort, Akhilesh Kumar, Jiuchun Zhang, Kimberley Smuga-Otto, James A. Thomson, and Igor I. Slukvin

Supplementary Experimental Procedures

Embryonic Stem Cells.

Rh366.4 and Rh456 ESCs were derived from in vivo-flushed blastocysts (Thomson et al., 1995; Thomson and Marshall, 1998).

Immunofluorescence

iPSCs were washed with PBS, fixed in 1% paraformaldehyde at 4°C for 30 mins, and permeabilized in 90% methanol for 30 mins at -20°C. Cells were incubated with primary antibodies Oct 3/4, Sox2 and Nanog (see Supplementary Table S1) at 1:200 dilution in PBS with 2% FBS overnight at 4°C. Following washing with saline, cells were incubated with secondary antibodies (either donkey anti-rabbit alexa fluor 488 (Life Technologies) or donkey anti-mouse alexa fluor 568 (Life Technologies) antibodies at a 1:500 dilution in PBS with 2% FBS for 1 hour at room temperature. Images were captured using the EVOS FL Auto cell imaging system (Life Technologies). Alkaline Phosphatase staining was completed according to the manufacturers instructions, VECTOR Blue Alkaline Phosphatase (AP) Substrate Kit (VECTOR laboratories SK-5300).

Hematopoietic Differentiation of NHP iPSCs in defined conditions.

The iPSCs were differentiated in feeder free and chemically defined conditions as described previously (Uenishi et al., 2014). Briefly, single cell suspensions of iPSCs were plated at a density of 2,500 cells/cm^2 onto six well plates coated with a mixture of 0.25μg/cm^2 each of ColIV and TenC in Primate ES medium (Reprocell) supplemented with 4ng/ml FGF2 and 10μM Rho Kinase inhibitor (Tocris Y-27632). After 24hrs, the medium was changed to IF9S medium supplemented with 4μM CHIR99021, 15ng/ml Activin A, 50ng/ml FGF2, 2mM LiCl, 50ng/ml VEGF and 1μM Rho kinase inhibitor. The medium was then changed to IF9S medium supplemented with 50ng/ml of VEGF and 50ng/ml of FGF2 on day 2. IF9S medium supplemented with 50 ng/ml FGF2, VEGF, TPO, SCF, IL-6, and 10 ng/ml IL-3 was used on day 4. On day 6, additional IF9S medium supplemented with the same six factors were added to the cultures without aspirating the old medium. Differentiation was conducted in a hypoxic conditions from day 0 to day 4, and then in a normoxia in the remaining days. Cells were dissociated with 1x TrypLE and collected for analysis.

RNA Extraction and Quantitative RT-PCR

RNA was extracted with Illustra RNAspin mini RNA isolation kit (GE Healthcare). Equal amounts of RNA was used for cDNA synthesis using Quantitect Reverse Transcription kit (Qiagen) . The mRNA levels of the indicated genes were analyzed in triplicates using Power SYBR Green PCR master mix (Applied Biosystems). The reactions were run on a Mastercycler RealPlex Thermal Cycler (Eppendorf) and the expression levels were calculated by minimal cycle threshold values (Ct) normalized to the reference expression of β actin. The primer sequences are listed in supplementary Table S2.

Supplementary References

Thomson, J.A., Kalishman, J., Golos, T.G., Durning, M., Harris, C.P., Becker, R.A., and Hearn, J.P. (1995). Isolation of a primate embryonic stem cell line. Proc Natl Acad Sci U S A *92*, 7844-7848.

Thomson, J.A., and Marshall, V.S. (1998). Primate embryonic stem cells. Curr Top Dev Biol *38*, 133-165.

Uenishi, G., Theisen, D., Lee, J.H., Kumar, A., Raymond, M., Vodyanik, M., Swanson, S., Stewart, R., Thomson, J., and Slukvin, I. (2014). Tenascin C promotes hematoendothelial development and T lymphoid commitment from human pluripotent stem cells in chemically defined conditions. Stem cell reports *3*, 1073-1084.

Supplementary Figures:

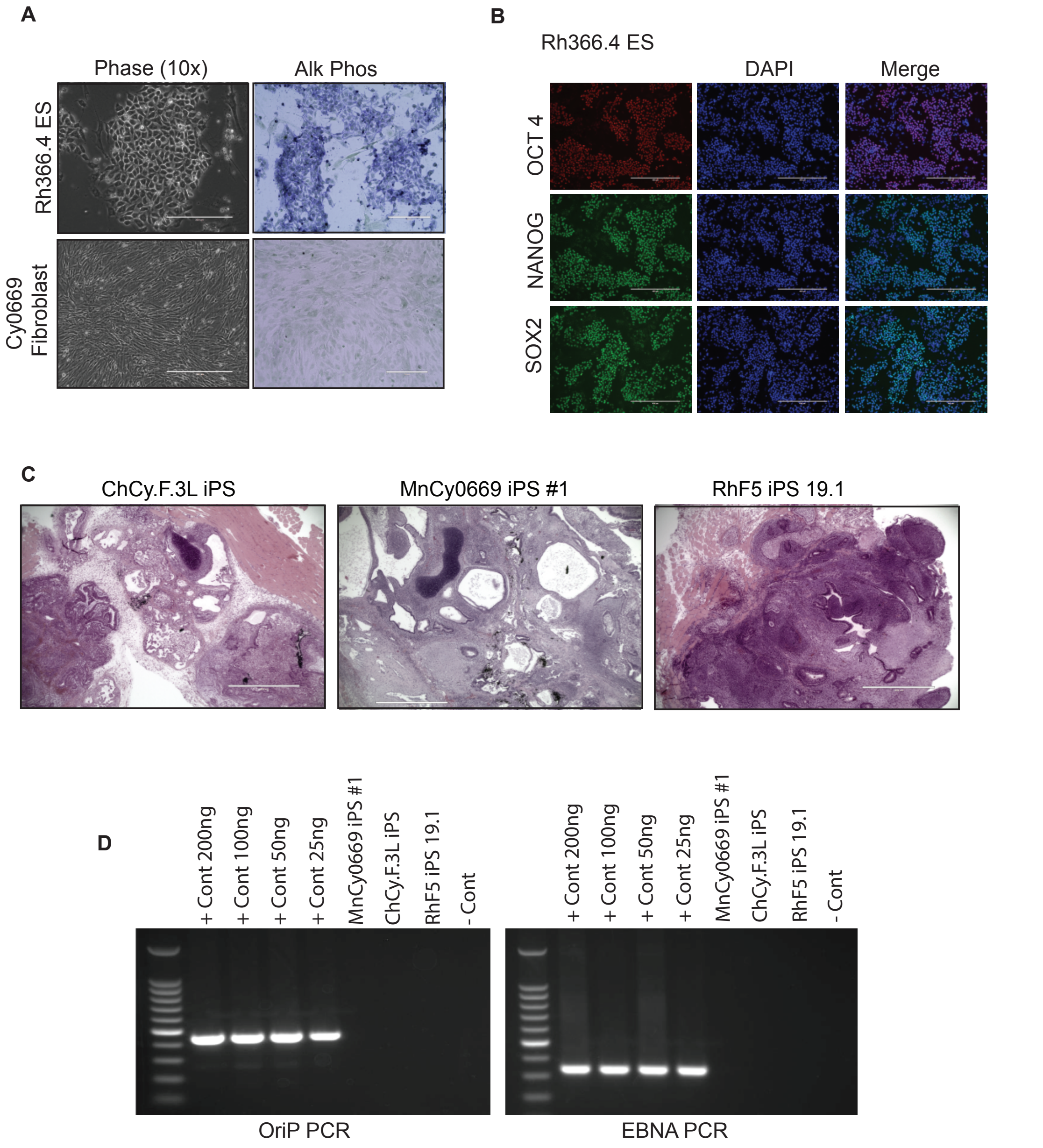

Supplementary Figure S1. Characterization of NHP iPSCs. (A) Morphology and alkaline phosphatase staining of rhesus ESCs. Fibroblasts from cynomolgus monkey served as a negative control. B) ES colonies from Rh366.4 were stained for pluripotency markers. (C) Low magnification images show teratoma formation by the indicated NHP iPSCs. (D) PCR analysis of iPSCs to confirm the absence of episomal reprogramming plasmids. Related to Figure 1.

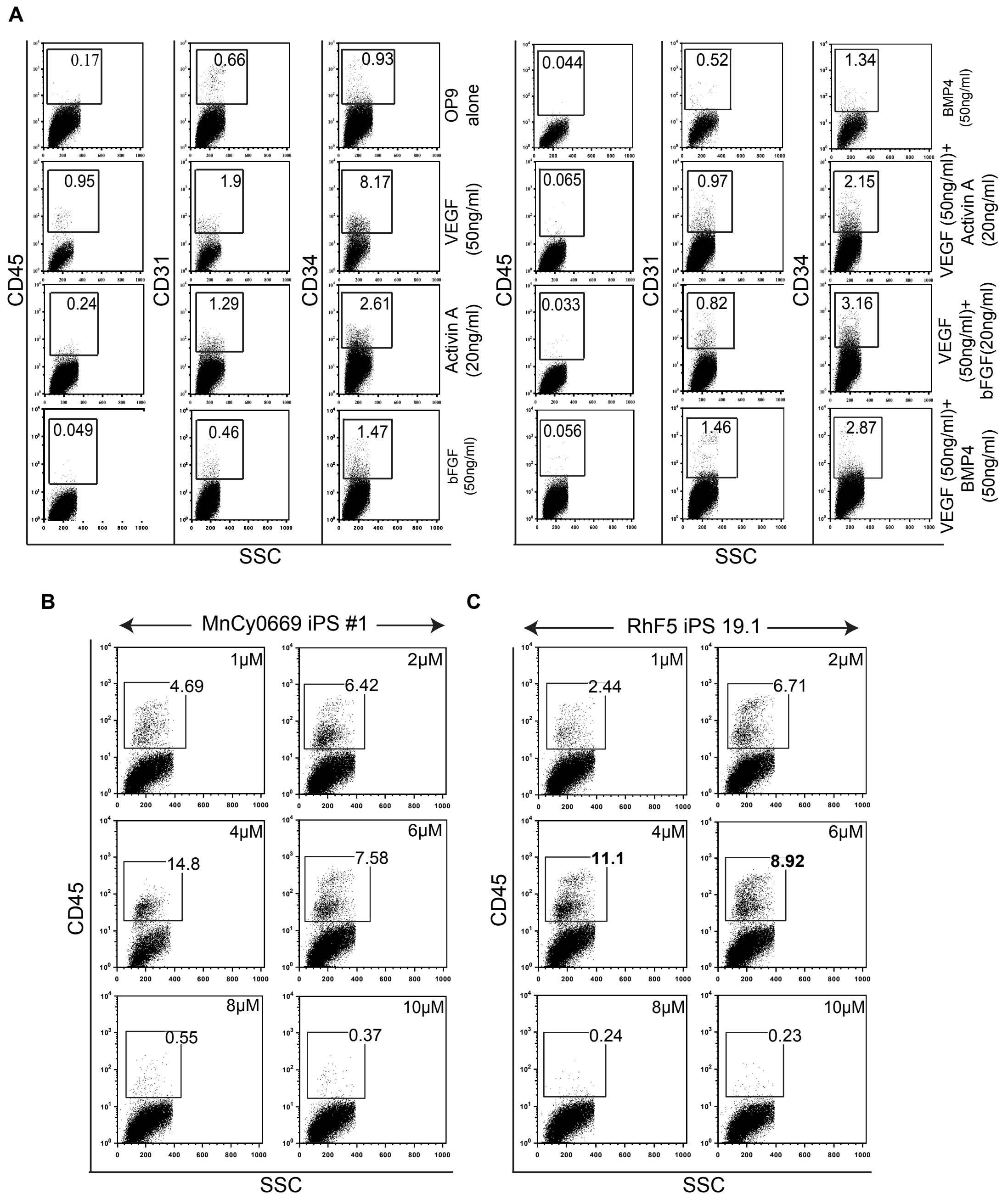

Supplementary Figure S2. Effect of various growth factors and CHIR99021 on hematopoiesis from NHP-iPSCs. (A) Comparative effect of different growth factor combinations on the generation of $CD45^{+}$ cells from MnCy0669 iPS#1 (B) Effect of different doses of CHIR99021 on hematopoietic differentiation of RhF5 iPS 19.1 and MnCy669 iPS#1. Related to Figure 2.

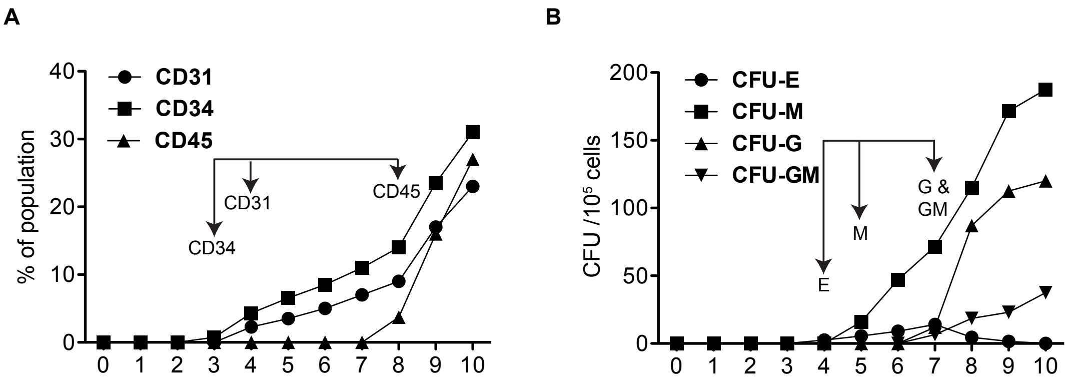

Supplementary Figure S3. (A) Percentages of the expression of CD45, CD34 and CD31 in Cy.F.3L iPSC/OP9 coculture following 10 days of differentiation as determined by flow cytometry. (B) Kinetics of different CFU types in Cy.F.3L iPSC/OP9 co-culture. Related to Figure 3.

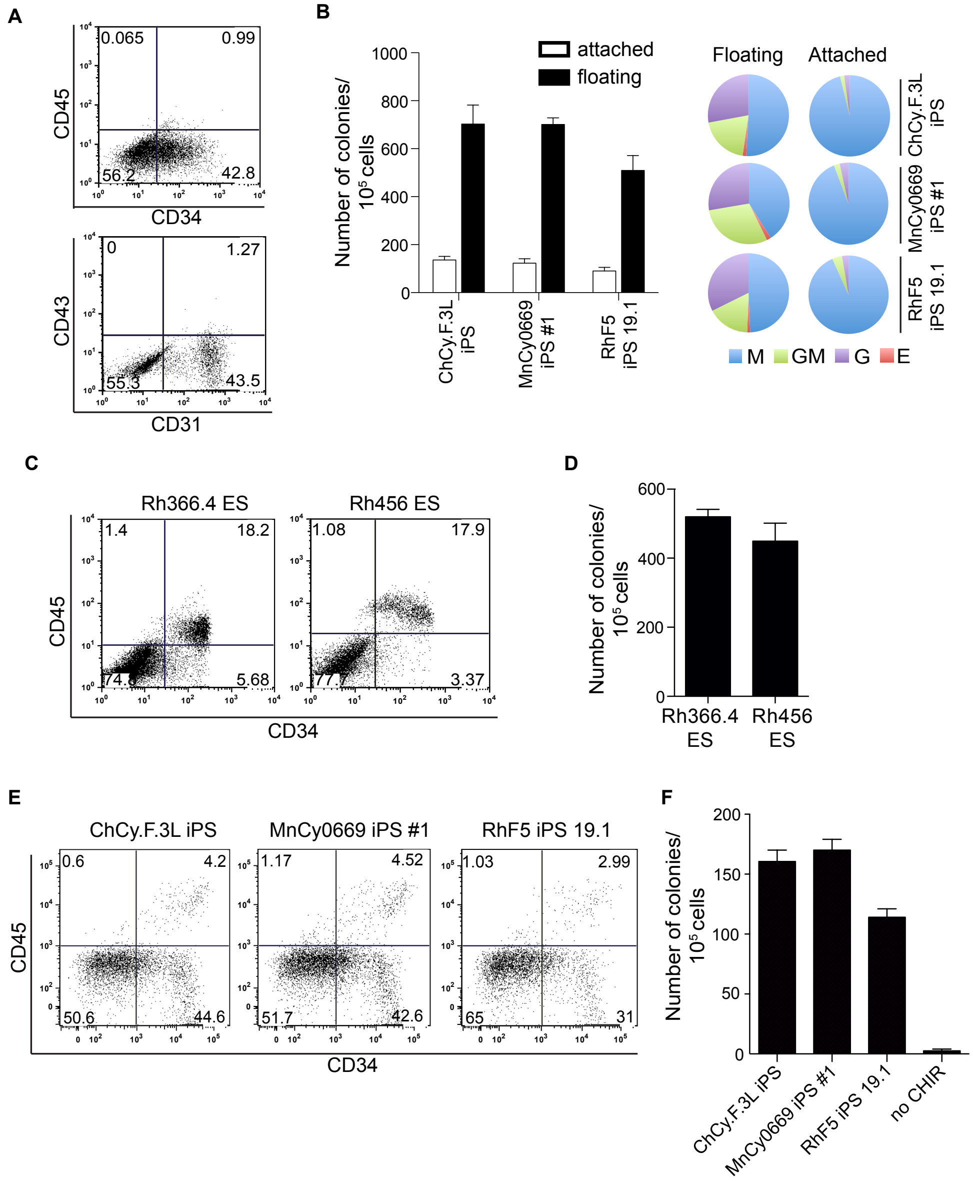

Supplementary Figure S4. Characterization of hematopoietic differentiation from NHP PSCs. (A) Flow cytometric analysis of the attached fraction after removal of floating cells on day 10 OP9-iPSC coculture. (B) CFU assay of the attached and floating fraction on day 10 of differentiation. Pie charts depict the relative proportions of CFU. Error bars are mean± SE from 3 independent experiments. (C) Rhesus ESCs Rh366.4 ES and Rh456 ES were differentiated on OP9 with CHR99021 and VEGF for 10 days and analyzed by flow cytometry. (D) CFU potential of the day 10 Rhesus ESCs differentiated on OP9 in presence of CHIR99021. Error bars are mean± SE from 3 independent experiments. (E) NHP iPSCs were differentiated on tenascin C and collagen IV coated plates in chemically defined conditions in the presence of CHIR99021 and VEGF (Uenishi et al., 2014). The cells were analyzed on day 8 of differentiation by flow cytometry. Cells differentiated without CHIR99021 gave rise to less than 0.5% of $CD45^+$ cells. (F) CFU analysis of NHP iPSCs differentiated in chemically defined conditions with CHIR99021. Typically, CFUs were not detected without CHIR99021, except few macrophage colonies in MnCy0669 iPS#1 (no CHIR bar). Error bars are mean± SE from 3 independent experiments. Related to Figure 3.

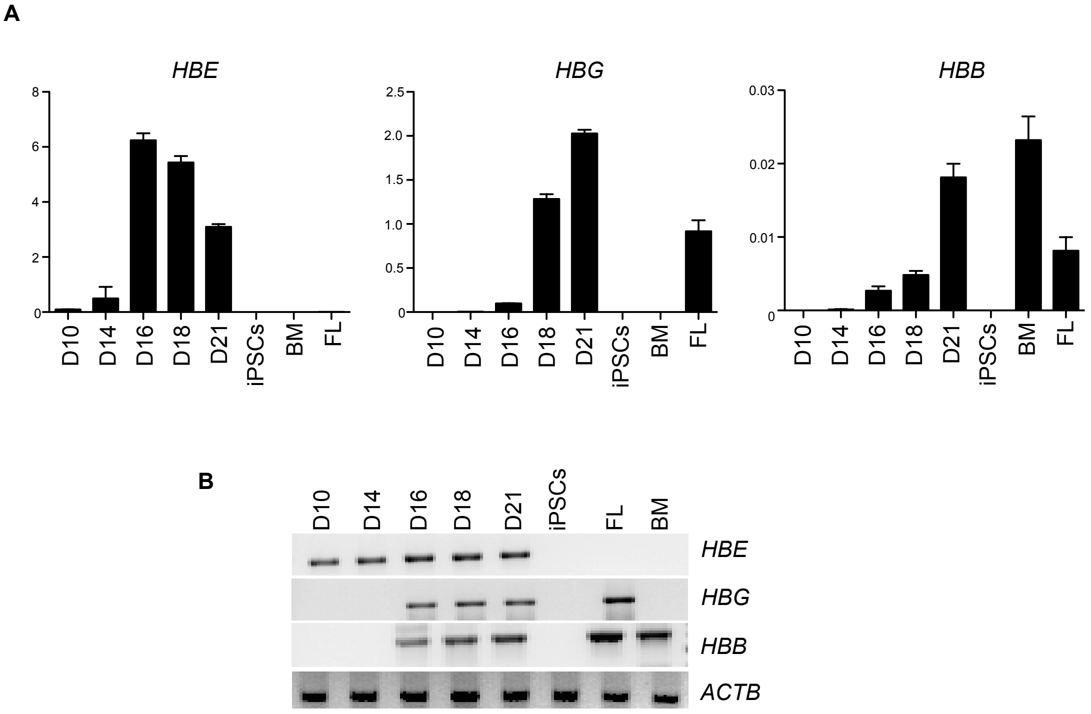

Supplementary Figure S5. Expression of embryonic ε globin (*HBE*), fetal γ globin (*HBG*) and adult β globin (*HBB*) in erythroid cultures from iPSCs. (A) qRT-PCR analysis of hemoglobins in cells cultures during erythroid differentiation. Relative expression normalized to β-actin (*ACTB*) is shown. Error bars are mean± SE from at least 3 experiments. BM (bone marrow) and FL (fetal liver) mononuclear cells were used as positive controls. (B) The PCR products were resolved on 1.5% agarose gel and visualized using ethidium bromide. Related to Figure 4.

Supplementary Table S1. List of Antibodies Used in Study, Related to Figures 1-5.

NAME	CLONE	COMPANY
Anti-NHP CD45	MB4-6D6<br>D058-1283	Miltenyi Biotech<br>BD- Biosciences
Anti-human CD34	563	BD- Biosciences
Anti-human CD-31	WM59	BD- Biosciences
Anti-human CD43	DFT-1	Acris Antibodies
Anti-human CD45RA	5H9	BD- Biosciences
Anti-human CD38	AT-1	StemCell Technologies
Anti-human CD90	5E10	BD- Biosciences
Anti-human APJ	72133	R&D Systems
Anti-human CD11b	ICRF44	BD- Biosciences
Anti-human CD71	L01.1	BD- Biosciences
Anti-human CD41a	HIP8	BD- Biosciences
Anti-human CD42a	ALMA.16	BD- Biosciences
Anti-human CD3ε	SP34	BD- Biosciences
Anti-human CD4	L200	BD- Biosciences
Anti-human CD5	UCHT2	Biolegend
Anti-human CD7	MT701	BD- Biosciences
Anti-rat TCRα/β	R73	Biolegend
Anti-human CD8	SK1	Biolegend
Anti-human CD56	B159	BD- Biosciences
Anti-human CD16	3G8	BD- Biosciences
Anti-human CD159a	Z199	Bekman Coulter
Anti-human Oct3/4	C10	Santa Cruz
Anti-human Nanog	D73G4	Cell Signaling
Anti-human Sox2	D6D9	Cell Signaling

Supplementary Table S2. Primer Sets Used in Study, Related to Figures 2 and 5 and Supplementary Figure S1

**GENE**	**Accession Numbers**	**PRIMER SEQUENCE**
KDR	XM_005555271.1	F: ATGCACGGCATCTGGGAATC<br>R: GTCACTGTCCTGCAAGTTGCTGTC
T	XM_001101421.2	F: GACAATTGGTCCAGCCTTG<br>R: GGGTACTGACTGGAGCTGGT
HBE	M81364.1	F:TGCATTTTACTGCTGAGGAGA<br>R:AAGAGAACTCAGTGGTACTT
HBG	M19433.1	F:CAGTTCCACACACTCGCTTCTGG<br>R:GTGATCTCTTAGCAGAATAGA
HBB	NM_001283367.1	F: ACACTTGCTTCTGACACAACTGT<br>R: ATTAGGCAGAATCCAGATCCTCA
RAG1	NM_000448.2	F: CCTGCTGAGCAAGGTACCTCA<br>R: ATCTGGGGCAGAACTGAGTCC
RAG2	XM_005578160.1	F: ACCTGGTTTAGCGGCAAAGA<br>R: TTTTGGGCCAGCCTTTTTGG
CD3E	AB583147.1	F: ACCTGTTCCCAACCCAGACT<br>R: GATCCTGCTGGCCTTTCCG
PRF1	XM_001107967.2	F: GAGGGGAGAGCACAAAGGAC<br>R: CGGATGTCCTCTCTTCACCG
IFNG	NM_001287657.1	F: TGACTCGAATGTCCAACGCA<br>R: CCCTATTTTAGCTGCTGGCG
EBNA	Custom designed	F: GAGGAACTGCCCTTGCTATT<br>R: CATCTCCATCACCTCCTTCATC
OriP	Custom designed	F: AGGCTACACCAACGTCAATC<br>R: GAGCACCTCACATACACCTTAC
ACTB	AY497558.1	F: GCAGGAGATGGCCACGGCGCC<br>R: TCTCCTTCTGCATCCTGTCGGC
