# Supplementary material for: GSK3β Inhibition Promotes Efficient Myeloid and Lymphoid Hematopoiesis from Non-human Primate-Induced Pluripotent Stem Cells
Source: Stem Cell Reports. 2016 Jan 21;6(2):243–56. doi: 10.1016/j.stemcr.2015.12.010 (PMC4750098; doi:10.1016/j.stemcr.2015.12.010)
Supplement: Document S2. Article plus Supplemental Information [file mmc2.pdf]

Stem Cell Reports
Resource
ISSCR
OPEN ACCESS

GSK3β Inhibition Promotes Efficient Myeloid and Lymphoid Hematopoiesis from Non-human Primate-Induced Pluripotent Stem Cells

Saritha S. D'Souza,[1] John Maufort,[1] Akhilesh Kumar,[1] Jiuchun Zhang,[2] Kimberley Smuga-Otto,[2] James A. Thomson,[2,3,4] and Igor I. Slukvin[1,3,5,*]
[1]National Primate Research Center, University of Wisconsin, 1220 Capitol Court, Madison, WI 53715, USA
[2]Morgridge Institute for Research, 309 North Orchard Street, Madison, WI 53715, USA
[3]Department of Cell and Regenerative Biology, School of Medicine and Public Health, University of Wisconsin, Madison, WI 53707, USA
[4]Department of Molecular, Cellular & Developmental Biology, University of California, Santa Barbara, CA 93106, USA
[5]Department of Pathology and Laboratory Medicine, University of Wisconsin, 1685 Highland Avenue, Madison WI 53705, USA
*Correspondence: islukvin@wisc.edu
http://dx.doi.org/10.1016/j.stemcr.2015.12.010
This is an open access article under the CC BY license (http://creativecommons.org/licenses/by/4.0/).

SUMMARY

Advances in the scalable production of blood cells from induced pluripotent stem cells (iPSCs) open prospects for the clinical translation of de novo generated blood products, and evoke the need for preclinical evaluation of their efficacy, safety, and immunogenicity in large animal models. Due to substantial similarities with humans, the outcomes of cellular therapies in non-human primate (NHP) models can be readily extrapolated to a clinical setting. However, the use of this model is hampered by relatively low efficiency of blood generation and lack of lymphoid potential in NHP-iPSC differentiation cultures. Here, we generated transgene-free iPSCs from different NHP species and showed the efficient induction of mesoderm, myeloid, and lymphoid cells from these iPSCs using a GSK3β inhibitor. Overall, our studies enable scalable production of hematopoietic progenitors from NHP-iPSCs, and lay the foundation for preclinical testing of iPSC-based therapies for blood and immune system diseases in an NHP model.

INTRODUCTION

Induced pluripotent stem cells (iPSCs) have created novel opportunities for the scalable manufacture of blood products for clinical use. Recent advances in hematopoietic differentiation from human pluripotent stem cells have brought the clinical translation of iPSC-derived blood products close to reality. Further progression requires proof-of-concept animal studies in addition to preclinical safety and toxicity assessment of stem cell therapies in animal models. Due to the significant differences in hematopoietic system homeostasis, cell surface markers, major histocompatibility complex (MHC) antigens, requirements for engraftment of hematopoietic cells (Harding et al., 2013; Trobridge and Kiem, 2010), and short life span, rodent models have a limited value for assessing the immunogenicity and safety of iPSC-derived therapies. Because humans and non-human primates (NHPs) share similar hematopoietic stem cell (HSC) dynamics, homing, and engraftment properties (reviewed in Trobridge and Kiem, 2010), orthologous MHC genes (Adams and Parham, 2001), and a very similar killer cell immunoglobulin-like receptor (KIR) structure and organization (Bimber et al., 2008; Parham et al., 2010), NHPs will be the most appropriate model to address the therapeutic efficacy and immunogenicity of allogeneic blood products. In addition, NHP models are critical for evaluating the long-term safety of stem cell therapies.

However, the use of an NHP model is hampered by the limited availability of clinically relevant NHP-iPSC lines. While the majority of NHP-iPSCs described in the literature were generated using retroviral vectors, human iPSCs intended for eventual therapeutic use need to be generated using transgene-free technologies. In addition, the efficiency of hematopoietic differentiation from NHP PSCs remains relatively low, and generation of lymphoid cells from them represents a significant challenge (Gori et al., 2012, 2015; Hiroyama et al., 2006; Shinoda et al., 2007; Umeda et al., 2004, 2006). Here, we describe generation of clinically relevant transgene-free iPSCs from different NHP species, including rhesus, Chinese cynomolgus, and Mauritian cynomolgus monkeys, and demonstrate that GSK3β inhibition is essential to induce rapid and efficient differentiation of the NHP-iPSCs into multipotential hematopoietic progenitors. NHP-iPSC-derived hematopoietic progenitors were capable of differentiating further into mature cell types of myeloid and lymphoid lineages, including natural killer (NK) and T cells. The kinetics and hierarchy of hematopoietic differentiation from NHP-iPSCs was similar to those of human PSCs. Overall, these studies lay the foundation for advancing an NHP model for preclinical testing of iPSC-based therapies for blood diseases.

RESULTS

Generation and Characterization of iPSCs from Rhesus, Chinese Cynomologus, and Mauritian Cynomologus Macaques

Primate fibroblasts were generated from skin punches of rhesus, Chinese cynomologus, and Mauritian macaques, then reprogrammed into iPSCs using EBNA/OriP-based episomal plasmids (Yu et al., 2009). Three to four weeks following electroporation of fibroblasts, iPSC colonies morphologically similar to both human and NHP embryonic stem cells (ESCs) began to appear. A subset of these colonies was picked and expanded on mouse embryonic fibroblasts (MEFs) and then transitioned to vitronectin-coated plates, where they were further expanded and characterized. iPSCs from all three NHP species grew as colonies morphologically similar to NHP ESCs and expressed the pluripotency factors OCT4, NANOG, and SOX2 (Figures S1A, S1B, 1A, and 1B). In addition, NHP-iPSCs stained positive for alkaline phosphatase similarly to ESCs (Figures 1B and S1A), formed teratomas following injection into the hind leg of SCID-beige mice (Figures 1C and S1C), and maintained a normal karyotype (Figure 1D). PCR analysis of iPSCs confirmed that they no longer contained the episomal reprogramming plasmids (Figure S1D). The established RhF5 iPS 19.1 line from rhesus macaque, the ChCy.F.3L iPS line from Chinese cynomolgus macaque, and the MnCy0669 iPS#1 line from Mauritian cynomolgus macaque were used for subsequent hematopoietic differentiation studies.

GSK3β Inhibition Promotes Mesoderm and Blood Formation from NHP-iPSCs

Previously, we established an OP9 co-culture system for the efficient differentiation of human PSCs, including iPSCs and ESCs (Choi et al., 2009a, 2009b; Vodyanik et al., 2005; Vodyanik and Slukvin, 2007). However, multiple attempts to apply this differentiation system to NHP-iPSCs failed to produce robust hematopoiesis. Analysis of expression of the mesodermal marker APLNR (D'Aniello et al., 2009; Vodyanik et al., 2010; Yu et al., 2012) in differentiation cultures revealed that inefficient hematopoiesis from NHP-iPSCs in OP9 co-cultures could be related to impaired induction of mesoderm, especially in cynomolgus iPSC cultures (Figure 2A). Thus, we tried to improve hematopoiesis by supplementing OP9 co-cultures with known factors that support mesoderm formation and hematopoietic specification from PSCs, including BMP4, basic fibroblast growth factor (bFGF), activin A, and vascular endothelial growth factor (VEGF). Although we observed an increase in CD34$^+$ and the formation of a limited number of CD45$^+$ cells following addition of VEGF, other factors had a negligible effect on hematopoiesis (Figure S2A).

Since the Wnt pathway has been shown to play a role in the induction of mesoderm and definitive hematopoiesis (Davis et al., 2008; Mendjan et al., 2014; Nostro et al., 2008; Sturgeon et al., 2014; Sumi et al., 2008), we tested whether mesoderm formation from NHP-iPSCs could be enhanced by using the GSK-3 inhibitor CHIR99021, a known Wnt agonist (Polychronopoulos et al., 2004). A dose-analysis study revealed that treatment with 4 μM of CHIR99021 on days 1 and 2 of differentiation coupled with continuous treatment with 50 ng/ml VEGF was optimal for the induction of CD45$^+$ hematopoietic cells and APLNR$^+$ mesoderm (Figures 2A, 2B, and S2B). The efficient induction of mesoderm in the presence of CHIR99021 was confirmed using qPCR. As shown in Figure 2C, CHIR99021-treated iPSCs from different NHP species expressed significantly higher levels of *T* and *KDR* mesodermal genes shortly after CHIR treatment.

Using CHIR and VEGF, we were able to induce blood production from rhesus, Chinese cynomolgus, and Mauritian cynomolgus monkey iPSCs (Figure 2D). The addition of stem cell factor (SCF), thrombopoietin (TPO) interleukin-3, (IL-3), and IL-6 hematopoietic cytokines further improved the output of hematopoietic progenitors in our differentiation system. When total cells were collected from differentiation cultures, the percentage of CD34$^+$CD45$^+$ cells from different primate species was approximately 20%–30% (Figure 2D). As in humans (Choi et al., 2009a; Vodyanik et al., 2006), the majority of hematopoietic progenitors induced from NHP-iPSCs co-expressed CD43 and CD31 (Figure 2D). Kinetic analysis of differentiation in OP9 co-cultures with CHIR99021 reveals striking similarities in hematopoietic differentiation between human iPSCs and NHP-iPSCs. Similar to human iPSC differentiation on OP9 (Vodyanik et al., 2005), the first hematoendothelial markers CD34 and CD31, were detected on day 4–5 followed by CD45, whose expression could be detected by day 8 (Figure S3A).

Based on this, we established the optimal differentiation protocol depicted in Figure 3A. To simplify the enrichment of hematopoietic progenitors, we collected only floating cells which became abundant at day 10 of differentiation of iPSCs in co-cultures with OP9. Flow cytometric analysis of day 10 floating cells revealed that more than 90% of them have the CD34$^+$CD45$^+$CD31$^+$CD38$^-$CD45RA$^-$ phenotype of multipotent hematopoietic progenitors (Figure 3B). In addition, the majority (>70%) of floating cells were CD90$^+$. Typically, we were able to produce 2–3 × 10^6 CD34$^+$CD45$^+$ floating cells from 10^6 iPSCs (Table 1). The attached fraction mainly consisted of CD34$^+$CD31$^+$ CD43$^-$CD45$^-$ endothelial cells (approximately 40%) and

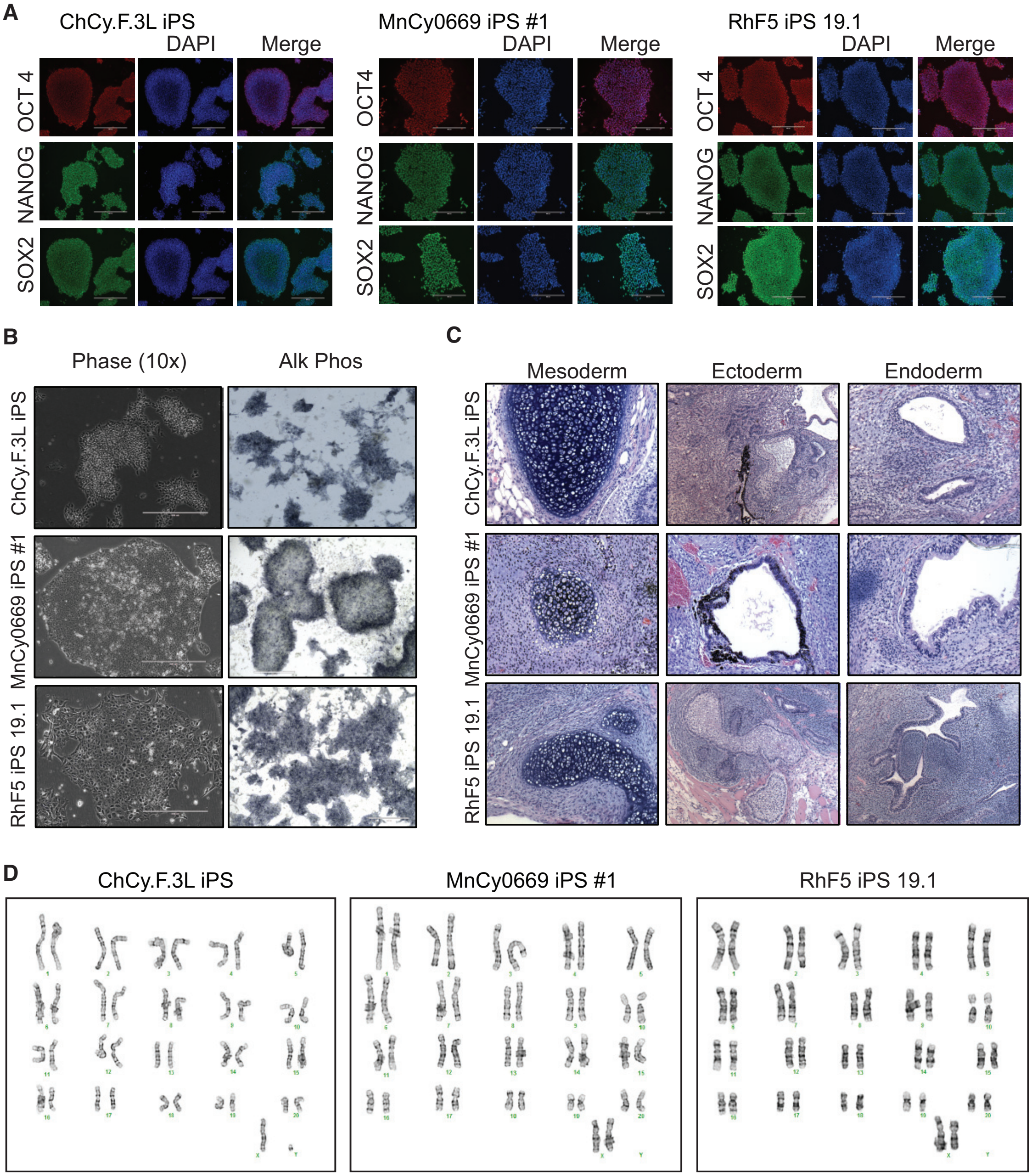

Figure 1. Generation and Characterization of Primate iPSCs

(A) Expression of pluripotency markers in RHF5-iPS 19.1 from rhesus, MnCy0669 iPS #1 from Mauritian cynomolgus, and ChCy.F3L from Chinese cynomolgus monkey iPSCs. ChCy.F.3L iPS photographs show colonies stained with mouse OCT3/4 and rabbit SOX2 antibodies followed by donkey anti-mouse Alexa Fluor 568 and donkey anti-rabbit Alexa Fluor 488 (upper and lower rows) and colonies stained with rabbit NANOG antibodies followed by anti-rabbit Alexa Fluor 488 (middle row). MnCy0669 iPS#1 and RhF5 iPS 19.1 photographs show colonies stained with mouse OCT3/4 and rabbit NANOG antibodies followed by donkey anti-mouse Alexa Fluor 568 and donkey anti-rabbit Alexa Fluor 488 (upper and middle rows) and colonies stained with rabbit SOX2 antibodies followed by anti-rabbit Alexa Fluor 488 (lower row). Scale bar represents 400 μm.

(B) Expression of alkaline phosphatase in NHP-iPSC lines. Scale bar represents 400 μm.

(C) Teratomas from the indicated NHP-iPSCs show derivatives of all three embryonic germline layers, including cartilage (mesoderm), retinal pigmented epithelium or keratinocytes (ectoderm), and gut-like structures (endoderm).

(D) Karyotypes of the generated NHP-iPSCs.

See also Figure S1.

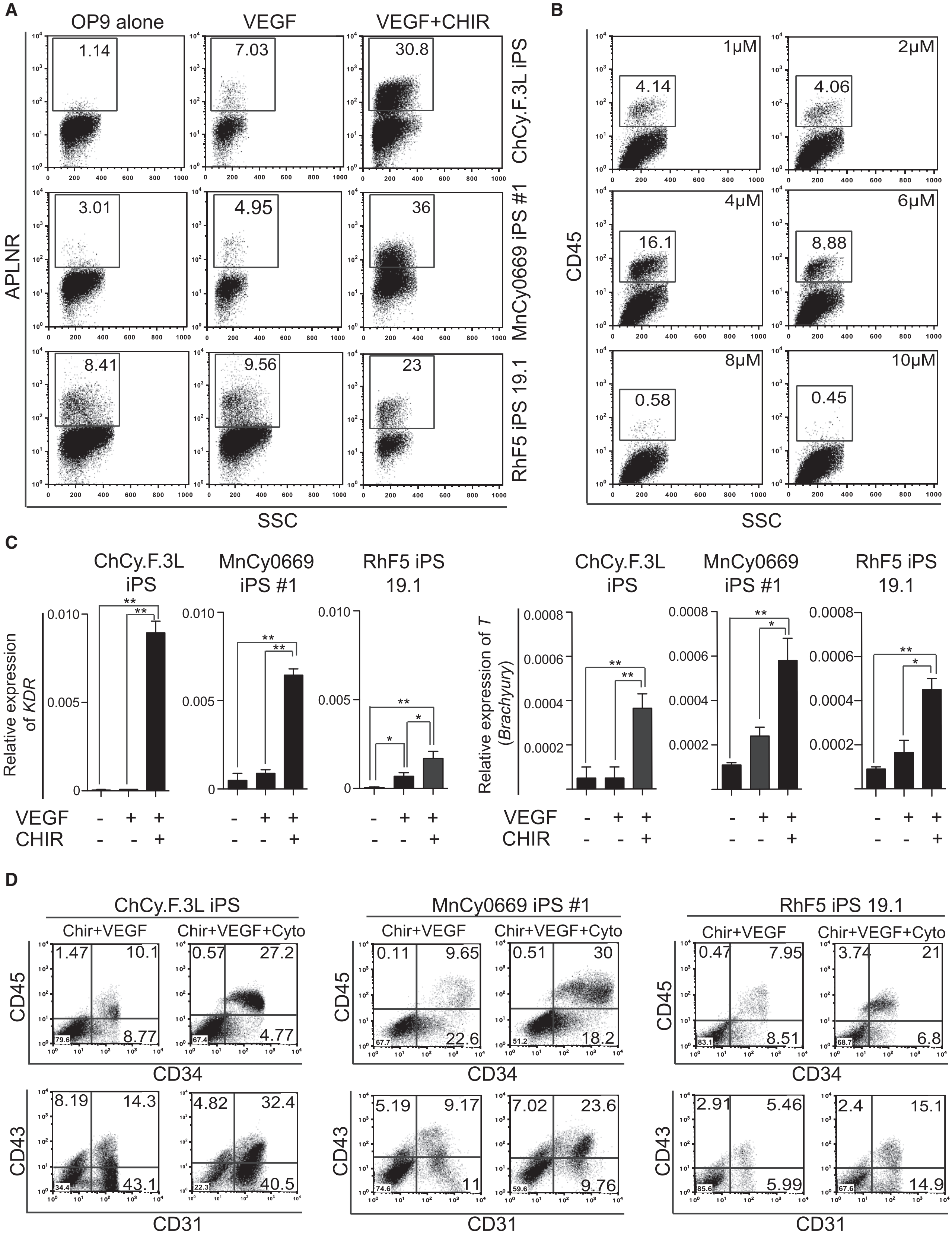

(legend on next page)

residual OP9 cells, with less than 2% being $CD45^+$ and $CD43^+$ hematopoietic cells (Figure S4A).

Next, we assessed the hematopoietic potential of cells collected on day 10 of differentiation using a colony-forming unit assay (CFU assay). In these studies, we used serum-containing MethoCult H4435 design for the detection of hematopoietic colonies from human somatic $CD34^+$ cells. The NHP-iPSC lines from all tested NHP species formed colonies in semisolid medium mainly consisting of CFU-M (-macrophage), CFU-GM (-granulocyte macrophage), and CFU-G (-granulocyte) (Figure 3C). The number of colonies was near 400–600 per 10^5 plated cells (Figure 3D), which is at least 8-fold higher than that reported for NHP ESCs and iPSCs (Abed et al., 2015; Gori et al., 2012, 2015; Shinoda et al., 2007; Umeda et al., 2006), and slightly less than the CFU numbers produced by rhesus cord blood $CD34^+$ cells. In total, we were able to generate 3.9–5.5 × 10^3 CFU from 10^6 iPSCs (Table 1). However, we observed a relatively higher proportion of CFU-M and CFU-G and a relatively lower proportion of multipotent CFU-GM and GEMM (granulocytes, erythrocytes, monocytes/macrophages, megakaryocytes) in iPSC-derived $CD34^+CD45^+$ cells compared with cord blood $CD34^+$ cells (Figure 3D). Despite the abundance of myeloid colonies in differentiation culture, we failed to detect a substantial number of erythroid colonies. Although we were able to improve detection of CFU-E from differentiated iPSCs by reducing fetal bovine serum to 10% in MethoCult, the number of erythroid colonies remained consistently low (Figure 3D). Kinetic analysis of the CFU potential during differentiation revealed that CFU-E were the first to appear at day 4 of differentiation followed by CFU-M on day 5, while both CFU-G and CFU-GM appeared on day 7 (Figure S3B). These kinetics resemble that of hematopoietic CFUs in human cultures (Vodyanik et al., 2005). The majority of colony-forming activity was found within the floating cell fraction, whereas the attached fraction generated a much lower number of colonies, most of which were CFU-M (Figure S4B).

To confirm that our differentiation protocol induces efficient blood formation from a broad range of NHP PSC lines, including NHP ESCs, we applied our protocol to induce hematopoietic differentiation from rhesus ESCs, R366.4 and R456. We, along with others, have previously reported that these cells produce mostly endothelial cells and very few (<1%) hematopoietic cells in conventional OP9 and embryoid body differentiation systems (Kaufman et al., 2004; Rajesh et al., 2007). Using our differentiation protocol with CHIR99021, we were able to induce a 17-fold higher production of $CD34^+CD45^+$ hematopoietic progenitors from these rhesus ESCs (Figure S4C) compared with prior studies. The number of colonies formed from rhesus ESCs in CFU assay was very similar to the number of colonies formed from differentiated NHP-iPSCs (about 500 per 10^5 plated cells; Figure S4D).

Recently, we developed chemically defined conditions for inducing hematopoiesis from human iPSCs (Uenishi et al., 2014). This protocol eliminates the variations associated with the use of xenogeneic feeder cells and serum. Although we failed to induce differentiation from NHP-iPSCs using the defined conditions described for human cells (Uenishi et al., 2014), the addition of CHIR99021 during the initial 2 days of differentiation in this system allowed for $CD34^+CD45^+$ hematopoietic progenitor formation and CFUs (Figures S4E and S4F), although hematopoietic differentiation of NHP-iPSCs in the defined system was less efficient when compared with OP9 co-culture. Thus, our findings indicate that GSK3β inhibition successfully allows for the induction of blood formation from a broad range of NHP PSCs, and could be applied to various differentiation protocols.

Differentiation of NHP-iPSCs toward Mature Cells of Myeloid Lineages

To confirm the potential of the day-10 hematopoietic progenitors to generate mature blood cells, we collected the floating cells, consisting mainly of $CD34^+CD45^+$ double-positive cells, and replated them on OP9 in differentiation medium containing 50 ng/ml SCF, 20 ng/ml TPO, 20 ng/ml IL-3, 20 ng/ml IL-6, and 0.5 U/ml erythropoietin (Figure 4A). Cytospins of the floating cells 3 days after replating showed abundant nucleated erythroid cells, granulocytes, and macrophages (Figure 4B). These findings were consistent among all tested NHP-iPSCs from different primate species. Interestingly, we observed numerous red blood

Figure 2. GSK3b Inhibition Promotes Mesoderm Formation and Efficient Hematopoiesis

(A) Effect of CHIR99021 and VEGF on the expression of APLNR on day 4 of differentiation of indicated iPSC lines in co-culture with OP9.

(B) ChCy.F.3L iPSCs in OP9 co-culture were treated with indicated doses of CHIR99021 along with 50 ng/ml VEGF for 2 days, and the amounts of CD45-positive cells were measured on day 10 by flow cytometry.

(C) The effect of 2 days of CHIR99021 treatment on the expression of *T* and *KDR* mesodermal genes as measured by qRT-PCR. Relative expression normalized to *ACTB* is shown. Error bars denote mean ± SE from at least three experiments (*$p < 0.01$, **$p < 0.001$).

(D) Effect of hematopoietic cytokines on blood production from NHP-iPSCs. iPSC lines from different NHP species were differentiated on OP9 with CHIR and VEGF with or without hematopoietic cytokines, and were analyzed on day 10 of differentiation by flow cytometry following collection of total cultures.

See also Figure S2.

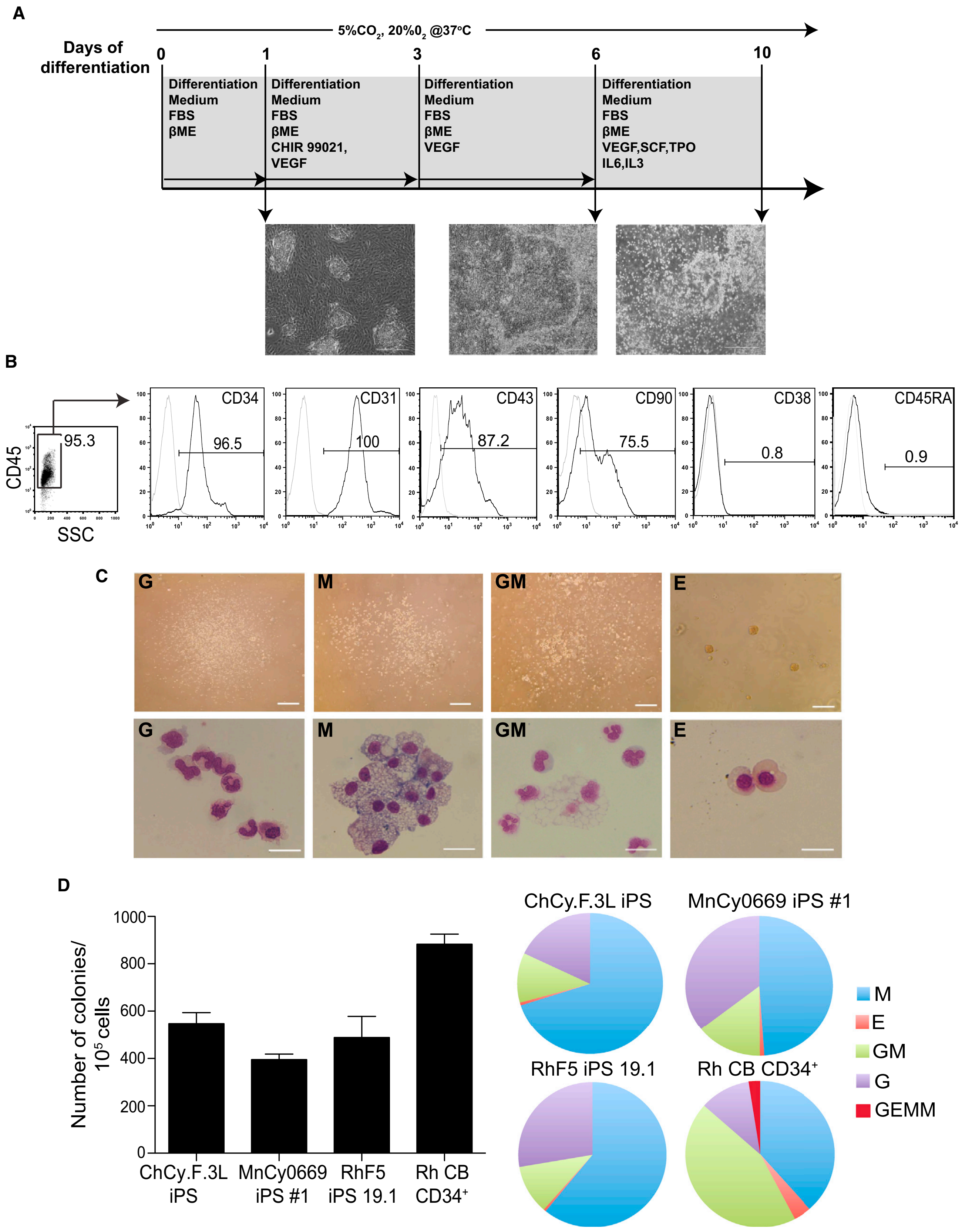

(legend on next page)

iPSCs	$CD45^+CD34^+$ Cells ($\times10^6$)	CFU ($\times10^3$)	Red Blood Cells ($\times10^6$)	Myeloid Cells ($\times10^6$)	$CD4^+CD8^+$ T Cells ($\times10^6$)	NK Cells ($\times10^6$)
MnCy0669 iPS#1	3.1 ± 0.2	3.9 ± 1.4	33.3 ± 8.8	12.7 ± 1.8	12.3 ± 1.4	3.5 ± 0.8
ChCy.F.3L iPS	2.8 ± 0.2	5.5 ± 2.7	17.7 ± 6.2	22.3 ± 1.5	10.7 ± 0.9	2.7 ± 0.6
RhF5 iPS 19.1	2.0 ± 0.2	5.1 ± 5.5	7.3 ± 0.9	8.1 ± 0.04	8.2 ± 0.4	1.0 ± 0.1

Table 1. Yield of Hematopoietic Cells from 1 × 10^6 iPSCs[a]

[a]Data shown are mean ± SE from three independent experiments.

cells in expansion cultures, despite relatively low CFU-E counts. Thus, we concluded that the low level of CFU-E is most likely an experimental artifact due to suboptimal conditions for their detection in a semisolid medium, rather than a reflection of an actual limited erythroid potential of hematopoietic cells generated from NHP-iPSCs. To confirm our conclusion and evaluate whether we can use our differentiation system to produce mature erythroid cells, we transferred day-10 hematopoietic progenitors onto OP9 and cultured them in the presence of TPO, SCF, and erythropoietin for an additional 7 days. As shown in Figure 4C, cultures of $CD34^+CD45^+$ cells in these conditions generated an almost pure population of $CD71^+$ $CD45^-$ red blood cells, most of which were enucleated. Typically, our cultures generated 1–2 × 10^7 red blood cells from 10^6 iPSCs (Table 1). Hemoglobin analysis by qRT-PCR revealed that erythroid cells during the early stages of expansion predominantly expressed embryonic ε- and fetal γ-hemoglobins with some adult β-hemoglobin (Figures S5A and S5B). However, as the expansion of these cells continues, ε-hemoglobin expression decreased while the expression of β-hemoglobin significantly increased, and at day 21 of differentiation the level of β-hemoglobin expression in erythroid cultures reached or exceeded its level in fetal liver and bone marrow mononuclear cells (Figures S5A and S5B).

To demonstrate megakaryocytic potential, we cultured day-10 floating cells with SCF, TPO, IL-3, IL-6, and IL-11 in feeder-free conditions. As shown in Figure 4C, these culture conditions generated megakaryocytic cells expressing CD41a and CD42a. Finally, we confirmed the potential of day-10 $CD34^+CD45^+$ cells to generate mature myelomonocytic cells by culturing them in the presence of GM-colony stimulating factor (GM-CSF) (Figure 4C). These myeloid cultures yielded 0.8–2.2 × 10^7 myelomonocytic cells from 10^6 iPSCs (Table 1).

Differentiation of NHP-iPSCs toward Lymphoid Lineages

As reported by the Keller group (Sturgeon et al., 2014), activation of β-catenin pathways using GSK3β inhibitor is critical for the generation of definitive hematopoietic cells with lymphoid potential from human PSCs. To find out whether GSK3β inhibition supports specification of hematopoietic progenitors with T lymphoid potential, we collected $CD45^+$ floating cells from day-10 differentiated cultures and replated them onto OP9 expressing DLL4 (OP9-DLL4) (Kennedy et al., 2012; Timmermans et al., 2009; Uenishi et al., 2014) as depicted in Figure 5A. By week 3, $CD4^+CD8^+$ double-positive T cells arose, which were also $CD5^+CD7^+$. In addition, we detected approximately 10% of $CD3^+TCR\alpha\beta^+$ double-positive cells in our T cell differentiation cultures (Figure 5B). We were typically able to obtain 8–12 × 10^6 of $CD4^+CD8^+$ cells from 10^6 iPSCs (Table 1). PCR analysis revealed greater expression levels of the *RAG1* and *RAG2* genes that are involved in the initiation of V(D)J recombination during B and T cell development in iPSC-derived T cells compared with peripheral blood T cells. Levels of *CD3E* expression were similar in T cells generated from iPSCs and isolated from peripheral blood (Figure 5C). To confirm T cell development, we analyzed the genomic DNA of the hematopoietic cells

Figure 3. Characterization of Hematopoiesis Induced in the Presence of GSK3β Inhibitor

(A) Schematic representation of the established differentiation protocol for induction of mesoderm and blood formation for the NHP-iPSCs.

(B) Phenotype of floating cells collected from MnCy0669#1 iPSC/OP9 co-culture on day 10 of differentiation.

(C) CFU potential of the floating cells. Microscopic images show colony morphology (upper panels) and cytospins (lower panels). Scale bar for CFU assay represents 200 μm and that for cytospins is 50 μm. G, granulocytes; M, macrophages; GM, granulocytes/macrophages; E, erythroid.

(D) Graphs display the frequency of colony-forming units (left) and the relative proportion of the different types of colony-forming units (right). Error bars denote mean ± SE from at least three experiments.

See also Figures S3 and S4.

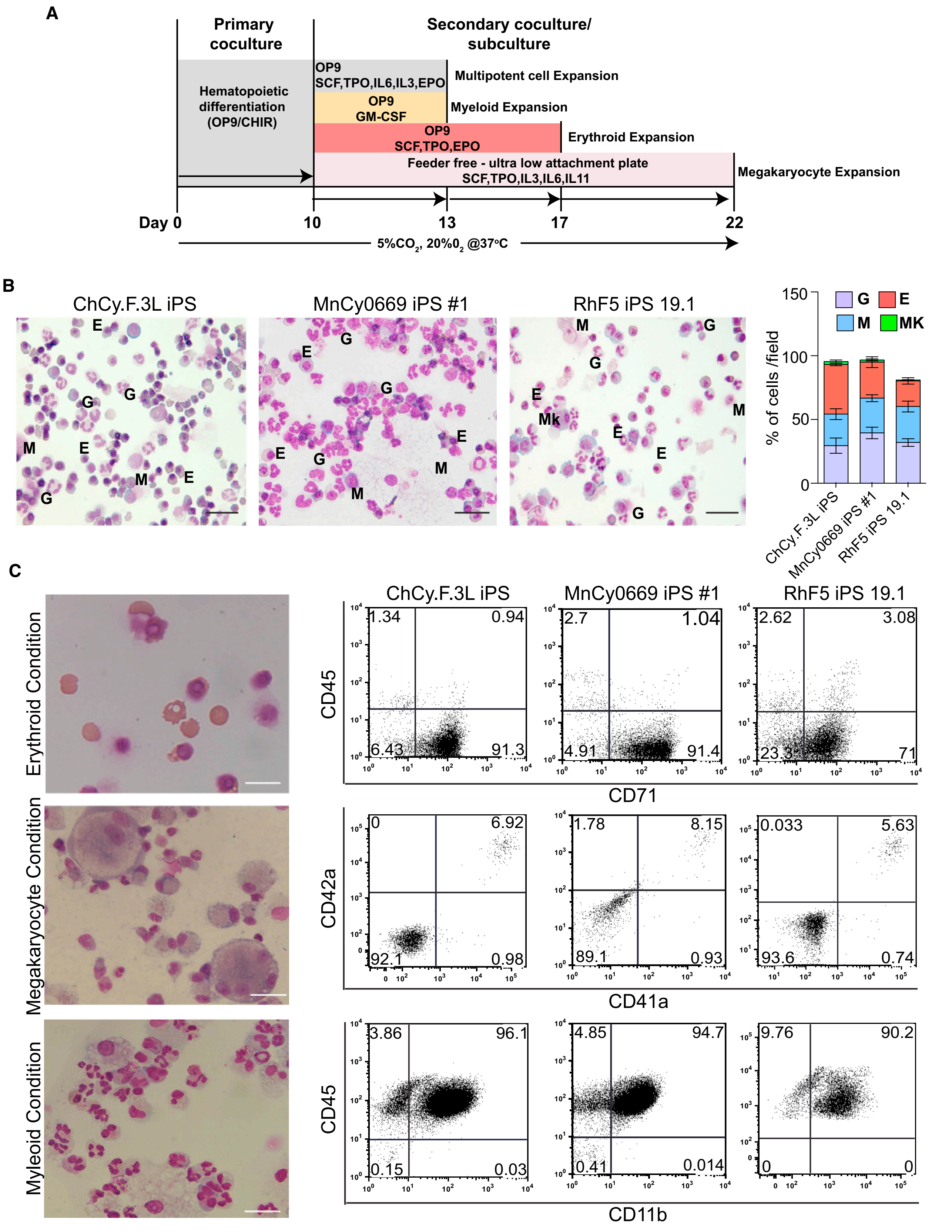

(legend on next page)

from OP9-DLL4 cultures for the presence of T cell receptor (TCR) rearrangements. This analysis demonstrated the presence of multiple PCR products of random V-J and D-J rearrangements at the β locus and V-J rearrangements at the γ locus, indicative of a polyclonal T lineage repertoire (Figure 5D).

To differentiate NK cells, floating cells from OP9 co-culture day 10 were isolated and cultured on confluent OP9-hDLL4 in the presence of IL-7, Flt3 ligand, and IL-2. Within approximately 2 weeks of the culture, lysis of OP9-hDLL4 stromal cells was observed. Flow cytometric analysis at 4 weeks using CD159a, a highly specific marker for NHP NK cells (Mavilio et al., 2005), along with CD56, confirmed the presence of NK cells in culture (Figure 5E). Similarly to peripheral blood NKs, iPSC-derived NK cells expressed *PRF1* perforin and *IFNG* genes (Figure 5F). Using our differentiation protocol we were able to obtain 1–3.5 × 10^6 NK cells from 1 × 10^6 iPSCs (Table 1).

DISCUSSION

This study presents a successful generation of transgene-free iPSCs from different primate species including rhesus, Chinese cynomolgus, and Mauritian cynomolgus monkeys using episomal vectors. The access to transgene-free NHP-iPSCs is critical for establishing clinically relevant large animal models that use cells analogous to human cells intended for clinical trials. In addition, we showed that GSK3β inhibition allows for the efficient production of hematopoietic progenitors with both myeloid and lymphoid potentials from NHP-iPSCs.

Several protocols have been described for the induction of hematopoietic differentiation from NHP-iPSCs. These protocols employed co-culture of monkey cells with OP9 or other feeders and an embryoid body method (Abed et al., 2015; Gori et al., 2012, 2015; Hiroyama et al., 2006; Shinoda et al., 2007; Umeda et al., 2004, 2006). However, these studies reported the generation up to 5% CD34$^+$ CD45$^+$ hematopoietic progenitors and up to 75 CFU per 10^5 cells in total cultures. In our studies, we demonstrated that the addition of GSK3β inhibitor potentiates mesoderm induction from NHP-iPSCs and increases the efficacy of clonogenic blood progenitor generation from NHP-iPSCs at least 10-fold, compared with previously published studies. In addition, hematopoietic commitment and differentiation in our system proceeded more rapidly, approximately within 6–10 days, compared with 15 or more days in other studies. Thus, our system simplifies the manufacture of blood cells for preclinical studies. Importantly, our differentiation system induces progenitors with T and NK cell lymphoid potentials. Overall, we were able to generate greater than 2 × 10^6 CD34$^+$CD45$^+$ multipotential hematopoietic progenitors containing greater than 4 × 10^3 CFU from 1 × 10^6 NHP-iPSCs in our differentiation system (Table 1). Recent studies have demonstrated hematopoietic engraftment in NOD/SCID/IL-2 receptor γ-chain-null mice following intrafemoral injection of differentiated NHP-iPSCs (Abed et al., 2015; Gori et al., 2015). Since our differentiation system makes it feasible to produce large numbers of CD34$^+$CD45$^+$ cells that are highly enriched in myeloid and lymphoid progenitors, it opens opportunities for preclinical testing of iPSC-derived blood products in a highly relevant NHP model.

Moving artificial blood products into the clinic requires proof-of-concept animal studies and preclinical safety and toxicity assessment of stem cell therapies in animal models before entering into clinical trials (FDA, 2008, 2013; Fink, 2009). Tumorigenicity, biodistribution, and immunogenicity are identified as areas of concern that need to be addressed through in vivo studies (Goldring et al., 2011; Sharpe et al., 2012). The Food and Drug Administration (FDA) considers two types of animal models acceptable for preclinical studies: evaluation of human cells in an immune compromised animal host and evaluation of analogous cells in a species-specific model (FDA, 2013). Both approaches have limitations for the assessment of tumorigenicity and biodistribution because human cells may behave differently in an animal host environment, and intrinsic differences could exist in cell properties between human and animal cells. However, analogous animal models would be a better predictor of immunogenicity and immunotoxicity, because they require immunocompetent hosts.

Figure 4. Generation of Mature Blood Cells from Day-10 CD34$^+$CD45$^+$ Hematopoietic Progenitors

(A) Schematic representation of the protocol followed for the production of myeloid, erythroid, and megakaryocytic cells from CD34$^+$CD45$^+$ hematopoietic progenitors derived from iPSCs in co-culture with OP9.

(B) Floating cells were collected on day-10 iPSC/OP9 co-culture, transferred on fresh OP9 cells, and cultured in differentiating medium with SCF, TPO, IL-3, IL-6, and erythropoietin. Cytospins of day-3 culture show that all three NHP lines produce all types of myeloid cells, including erythroid (E), granulocytes (G), monocytes/macrophages (M), and megakaryocytes (Mk). Scale bar represents 100 μm. Bar graph depicts the relative proportion of blood cells in cytospins.

(C) Directed differentiation of day-10 CD34$^+$CD45$^+$ floating cells to erythroid, megakaryocytic, and myeloid cells. Representative cytospins and flow cytometry plots are shown. Scale bar represents 50 μm.

See also Figure S5.

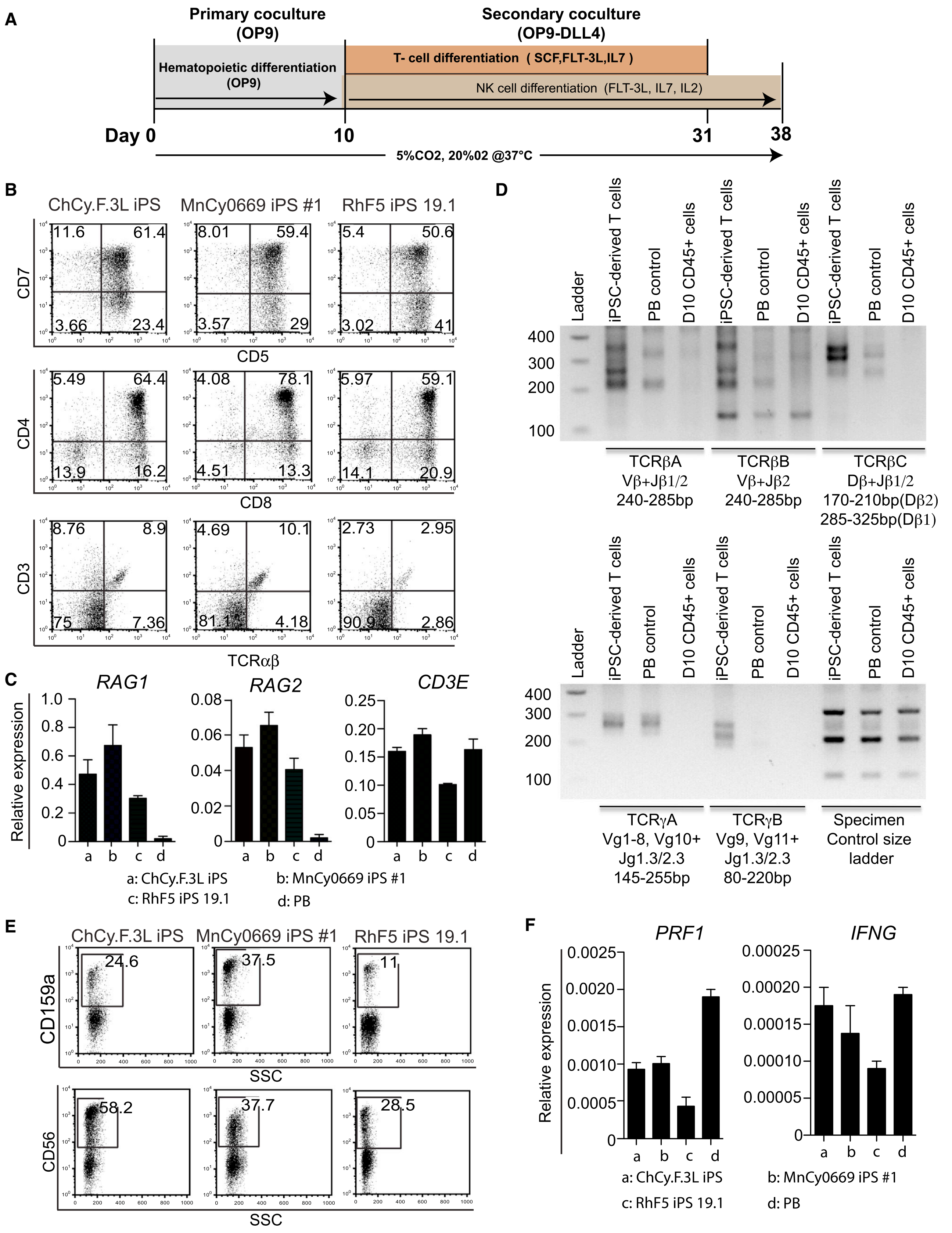

(legend on next page)

Although the FDA considers analogous cell product data an acceptable option for preclinical studies, it requires substantial similarity between analogous animal and human products (FDA, 2013). Due to the significant differences in the innate and adaptive immune system (Mestas and Hughes, 2004), hematopoietic system homeostasis, cell surface markers, and the requirements for hematopoietic cell engraftment (Harding et al., 2013; Trobridge and Kiem, 2010), rodent models are unlikely to fulfill this FDA requirement. The NHP model will be able to overcome these inherent limitations of rodent models, especially limitations related to completely different structure and MHC binding specificity between mouse and human KIRs (Natarajan et al., 2002; Parham et al., 2010). Because tumorigenicity associated with cell therapies may manifest several years after stem cell injection (Amariglio et al., 2009), NHP models would be critical in evaluating the long-term safety of stem cell therapies. The utility of an NHP model for safety evaluation can be appreciated from the lessons learned from gene therapy studies. The assumption about the safety of replication-incompetent retroviral vectors for gene therapy made in small animal studies was proved wrong in gene therapy trials targeting HSCs in children with X-linked severe combined immunodeficiency, who later developed vector-related leukemia several years after treatment with retrovirally modified stem cells (Hacein-Bey-Abina et al., 2003a, 2003b). Evidence that such retroviral-mediated gene therapy targeting HSCs can cause potential leukemia or malignant transformation has subsequently emerged from studies using NHPs (Seggewiss et al., 2006; Zhang et al., 2008).

In summary, our studies generated transgene-free iPSCs from various NHP species, and demonstrated the critical role of GSK3β inhibition for establishing a reproducible protocol for the efficient induction of mesoderm and myelolymphoid hematopoietic cells from NHP-iPSCs. This study lays the foundation for advancing an NHP model for preclinical testing of iPSC-based therapies for blood and immune system diseases.

EXPERIMENTAL PROCEDURES

Reprogramming NHP Fibroblast Cells

All animal procedures were approved by the University of Wisconsin Medical School's Animal Care and Use Committee. NHP fibroblast cells were reprogrammed with combinations of oriP/EBNA1-based episomal vectors described in Yu et al. (2009). RhF5-iPS 19.1, Cy.F 3L iPS, and Cy0669 iPS #1 cells were generated from fibroblasts isolated from a skin punch biopsies from a rhesus monkey, Chinese cynomolgus monkey, and Mauritian cynomolgus monkey, respectively. RhF5 19.1 and Cy.F 3L were reprogrammed with episomal combination #19 (Yu et al., 2009) using l-myc instead of c-myc. Cy0669 fibroblasts were reprogrammed using episomal combination #6 (Yu et al., 2009). For each reprogramming, 1–3 × 10^6 fibroblasts were transfected with 15 μg of each plasmid using the electroporator (250 V, 1000 μF, ∞ resistance; Bio-Rad) and seeded onto MEFs in 10-cm dishes. Cells were cultured in DMEM with 20% fetal bovine serum (FBS) and non-essential amino acids for 4–5 days. Medium was changed every 2 days. The cultures were split 1:3 if they became overconfluent before the 4–5 days. The cultures were then switched to E7 + sodium butyrate (DF3S with 100 ng/ml bFGF, 1.74 ng/ml transforming growth factor β, 10.7 μg/ml transferrin, 10 μg/ml insulin, and 0.1 mM sodium butyrate) until morphology changes began to appear, at which point the medium was switched over to primate iPSC medium (E8 with 100 ng/ml Nodal, 1.94 ng/ml glutathione, 1× Glutamax [Life Technologies], and 1× Chemically Defined Lipid Concentrate [Life Technologies]). Colonies began to appear within 3–4 weeks and were subsequently seeded onto MEF plates for expansion and characterization.

Teratoma Formation Assay

To test the developmental potential, NHP-iPSC lines were grown on 10-cm plates until approximately 80% confluent. Cells were then harvested with 0.5 mM EDTA and washed off the plate using maintenance medium. For each 10-cm dish, cells were resuspended in 400 μl of primate medium +220 μl of Matrigel. Volumes of 300, 200, and 100 μl from each cell line were injected into the hindlimb muscles of 6-week-old immunocompromised SCID-beige mice. After 6–10 weeks, teratomas were dissected and fixed in 4% paraformaldehyde. Samples were embedded in paraffin and processed with H&E staining at the Histology Lab at the School of Veterinary Medicine, University of Wisconsin-Madison.

Figure 5. Lymphoid Potential of NHP-iPSC Hematopoietic Progenitors Generated in Co-culture with OP9

(A) Schematic representation of protocol for lymphoid differentiation.

(B) Flow cytometric profile of T cells generated after 3 weeks of culture of $CD34^+CD45^+$ on OP9-DLL4 from the indicated NHP-iPSCs.

(C) Analysis of *RAG1, RAG2*, and *CD3E* gene expression by qRT-PCR. Relative expression normalized to β-actin is shown. Error bars denote mean ± SE from at least three experiments.

(D) Analysis of TCR rearrangement by genomic PCR in T cells obtained from the MnCy0669 iPS#1 line. The PCR products were resolved on 2% agarose gel and visualized using ethidium bromide. PB is peripheral blood (positive control) and D10 $CD45^+$ cells are floating cells collected from day-10 OP9 co-culture (negative control).

(E) Expression of CD56 and CD159a in NK differentiation cultures.

(F) Analysis of *PRF1* and *IFNG* gene expression by qRT-PCR. Relative expression normalized to *ACTB* is shown. Error bars denote mean ± SE from at least three experiments.

iPSC Differentiation in OP9 Co-culture

All iPSC lines were maintained in an undifferentiated state in primate ESC medium (ReproCell, Japan) supplemented with 4ng/ml of FGF2 by passages on MEFs every 3 days. A mouse OP9 stromal cell line was kindly provided by Dr. Toru Nakano (Osaka University). OP9 was maintained on gelatin-coated plastic in α-minimal essential medium (αMEM; Gibco-Invitrogen) supplemented with 20% defined FBS (HyClone Laboratories).

iPSCs prepared in suspension of small cell aggregates were added to OP9 cells in αMEM supplemented with 10% FBS (HyClone Laboratories) and 50 μM β-mercaptoethanol. On day 1 of co-culture, 4 μM CHIR99021 (Tocris) and 50 ng/ml VEGF (Peprotech) were added. The medium was changed on day 3 to fresh medium with 50 ng/ml VEGF. From day 6 of co-culture, a hematopoietic cytokine cocktail consisting of 50 ng/ml SCF, 20 ng/ml TPO, 20 ng/ml IL-6, and 20 ng/ml IL-3 (Peprotech) was added to the medium. iPSC/OP9 co-cultures were incubated for up to 10 days under standard conditions (37°C, 5% CO_2, ≥95% humidity). Half the medium was replaced with fresh medium on days 6 and 8. Floating blood cells or total cultures were collected for analysis. For total culture harvesting, hESC/OP9 co-cultures were dispersed by successive enzymatic treatment with collagenase IV (Gibco-Invitrogen; 1 mg/ml) for 20 min at 37°C, and 0.05% trypsin/0.5 mM EDTA (Gibco-Invitrogen) for 15 min at 37°C. Cells were resuspended by pipetting, washed twice with PBS containing 5% FBS, filtered through a 70-μm cell strainer (Becton Dickinson), and used for downstream analysis. For mesoderm induction, day-4 differentiated cells were analyzed by flow cytometry for expression of APLNR. RNA was isolated from day-4 differentiated cells after depleting OP9, cDNA was transcribed, and the relative expression of mesoderm genes KDR and T were analyzed by qRT-PCR.

Secondary Co-culture

For secondary co-culture, floating cells from day-10 iPSC-OP9 coculture were harvested and 2×10^5 cells were seeded onto fresh OP9 in differentiating medium in the presence of 50 ng/ml SCF, 20 ng/ml TPO, 20 ng/ml IL-3, 20 ng/ml IL-6, and 0.5 U/ml erythropoietin (R&D Systems). Floating cells were harvested after 3 days and analyzed by Wright-Giemsa staining. For myeloid expansion, day-10 floating cells were recultured on fresh OP9 monolayer in differentiating medium with 100 ng/ml GM-CSF. Flow cytometry was performed after 3 days. For erythroid differentiation, day-10 floating cells were seeded onto fresh OP9 in differentiating medium with 50 ng/ml SCF, 20 ng/ml TPO, and 0.5 U/ml erythropoietin for 7 days and analyzed by flow cytometry. For megakaryocyte differentiation, day-10 floating $CD34^+CD45^+$ cells were seeded onto ultralow attachment plates (Corning) in differentiating medium containing 50 ng/ml SCF, 20 ng/ml TPO, 20 ng/ml IL-3, 20 ng/ml IL-6, and 20 ng/ml IL-11.

Lymphoid Differentiation of Day-10 Cultures

The OP9 cell line expressing human DLL4 (OP9-DLL4) was established by using lentivirus expressing human DLL4 under the EF1α promoter. For T cell differentiation, the floating $CD45^+$ cells were collected from day-10 human PSC/OP9 co-culture, strained through a 70-μm cell strainer (BD), resuspended in T cell differentiation medium consisting of αMEM (Gibco) supplemented with 20% FBS (Hyclone), 5 ng/ml IL-7, 5 ng/ml Flt3 ligand, and 10 ng/ml SCF, and cultured on OP9-DLL4 for 3 weeks with weekly passage. Floating cells were collected for flow analysis and genomic DNA extraction for TCR rearrangement analysis using a TCR rearrangement kit (Invivoscribe Technologies). For NK cell differentiation, floating $CD45^+$ cells from iPSCs/OP9 co-culture were collected on day 10 and then co-cultured on OP9-DLL4 in αMEM containing 20% FBS, 20 ng/ml IL-7, 10 ng/ml Flt3 ligand, and 10 ng/ml IL-2 for up to 4 weeks at 37°C and 5% CO_2. Expression of NK cell-specific markers were then analyzed by flow cytometry.

Flow Cytometry

Cell surface staining was completed using antibodies that cross-reacted with cynomolgus and rhesus monkey cells coupled with 7-aminoactinomycin D for dead cell exclusion. The antibodies are listed in Table S1. Control staining with the appropriate isotype-matched mouse monoclonal antibody controls was included to establish a threshold for positive staining and subset gating. Samples were analyzed using a FACSCalibur flow cytometer (BD) and FlowJo software (Tree Star).

Colony Formation Assay

Cells were cultured in a 35-mm dish with 3 ml of enriched MethoCult (H4435; Stem Cell Technologies) with high serum concentration for 10–14 days, and individual colonies were scored by macroscopic morphology. Representative colonies were picked up and analyzed by microscopic morphology after Wright-Giemsa staining. In some experiments, the cells were cultured in serum-free MethoCult (H4436; Stem Cell Technologies) supplemented with either 10% or 15% FBS.

Statistical Analysis

Statistical analysis was performed using GraphPad Prism version 5 software. Data obtained from multiple experiments were reported as the mean ± SE. Student's t test and ANOVA were used to compare between groups. Differences were considered significant at $p < 0.01$.

SUPPLEMENTAL INFORMATION

Supplemental Information includes Supplemental Experimental Procedures, five figures, and two tables and can be found with this article online at http://dx.doi.org/10.1016/j.stemcr.2015.12.010.

ACKNOWLEDGMENTS

This work was supported by funds from the National Institute of Health (P51 OD011106, U01HL099773, UL1TR000427) and The Charlotte Geyer Foundation. We thank Toru Nakano at Osaka University Japan for providing the OP9 bone marrow stromal cell line, and Mathew Raymond for editorial assistance.

Received: July 17, 2015
Revised: December 18, 2015
Accepted: December 20, 2015
Published: January 21, 2016

REFERENCES

Abed, S., Tubsuwan, A., Chaichompoo, P., Park, I.H., Pailleret, A., Benyoucef, A., Tosca, L., De Dreuzy, E., Paulard, A., Granger-Locatelli, M., et al. (2015). Transplantation of *Macaca* cynomolgus iPS-derived hematopoietic cells in NSG immunodeficient mice. Haematologica *100*, e428–e431.

Adams, E.J., and Parham, P. (2001). Species-specific evolution of MHC class I genes in the higher primates. Immunol. Rev. *183*, 41–64.

Amariglio, N., Hirshberg, A., Scheithauer, B.W., Cohen, Y., Loewenthal, R., Trakhtenbrot, L., Paz, N., Koren-Michowitz, M., Waldman, D., Leider-Trejo, L., et al. (2009). Donor-derived brain tumor following neural stem cell transplantation in an ataxia telangiectasia patient. PLoS Med. *6*, e1000029.

Bimber, B.N., Moreland, A.J., Wiseman, R.W., Hughes, A.L., and O'Connor, D.H. (2008). Complete characterization of killer Ig-like receptor (KIR) haplotypes in Mauritian cynomolgus macaques: novel insights into nonhuman primate KIR gene content and organization. J. Immunol. *181*, 6301–6308.

Choi, K., Yu, J., Smuga-Otto, K., Salvagiotto, G., Rehrauer, W., Vodyanik, M., Thomson, J., and Slukvin, I. (2009a). Hematopoietic and endothelial differentiation of human induced pluripotent stem cells. Stem Cells *27*, 559–567.

Choi, K.D., Vodyanik, M.A., and Slukvin, I.I. (2009b). Generation of mature human myelomonocytic cells through expansion and differentiation of pluripotent stem cell-derived lin-CD34+CD43+CD45+ progenitors. J. Clin. Invest. *119*, 2818–2829.

D'Aniello, C., Lonardo, E., Iaconis, S., Guardiola, O., Liguoro, A.M., Liguori, G.L., Autiero, M., Carmeliet, P., and Minchiotti, G. (2009). G protein-coupled receptor APJ and its ligand apelin act downstream of Cripto to specify embryonic stem cells toward the cardiac lineage through extracellular signal-regulated kinase/p70S6 kinase signaling pathway. Circ. Res. *105*, 231–238.

Davis, R.P., Ng, E.S., Costa, M., Mossman, A.K., Sourris, K., Elefanty, A.G., and Stanley, E.G. (2008). Targeting a GFP reporter gene to the MIXL1 locus of human embryonic stem cells identifies human primitive streak-like cells and enables isolation of primitive hematopoietic precursors. Blood *111*, 1876–1884.

FDA. (2008). Cellular Therapies Derived from Human Embryonic Stem Cells—Considerations for Pre-clinical Safety Testing and Patient Monitoring, http://www.fda.gov/ohrms/dockets/ac/08/briefing/2008-0471B1_1.pdf.

FDA. (2013). Guidance for Industry: Preclinical Assessment of Investigational Cellular and Gene Therapy Products. IV. Recommendations for Investigational Cell Therapy (CT) Products, http://www.fda.gov/BiologicsBloodVaccines/GuidanceComplianceRegulatoryInformation/Guidances/CellularandGeneTherapy/ucm376136.htm.

Fink, D.W., Jr. (2009). FDA regulation of stem cell-based products. Science *324*, 1662–1663.

Goldring, C.E., Duffy, P.A., Benvenisty, N., Andrews, P.W., Ben-David, U., Eakins, R., French, N., Hanley, N.A., Kelly, L., Kitteringham, N.R., et al. (2011). Assessing the safety of stem cell therapeutics. Cell Stem Cell *8*, 618–628.

Gori, J.L., Chandrasekaran, D., Kowalski, J.P., Adair, J.E., Beard, B.C., D'Souza, S.L., and Kiem, H.P. (2012). Efficient generation, purification, and expansion of CD34(+) hematopoietic progenitor cells from nonhuman primate-induced pluripotent stem cells. Blood *120*, e35–e44.

Gori, J.L., Butler, J.M., Chan, Y.Y., Chandrasekaran, D., Poulos, M.G., Ginsberg, M., Nolan, D.J., Elemento, O., Wood, B.L., Adair, J.E., et al. (2015). Vascular niche promotes hematopoietic multipotent progenitor formation from pluripotent stem cells. J. Clin. Invest. *125*, 1243–1254.

Hacein-Bey-Abina, S., von Kalle, C., Schmidt, M., Le Deist, F., Wulffraat, N., McIntyre, E., Radford, I., Villeval, J.L., Fraser, C.C., Cavazzana-Calvo, M., et al. (2003a). A serious adverse event after successful gene therapy for X-linked severe combined immunodeficiency. N. Engl. J. Med. *348*, 255–256.

Hacein-Bey-Abina, S., Von Kalle, C., Schmidt, M., McCormack, M.P., Wulffraat, N., Leboulch, P., Lim, A., Osborne, C.S., Pawliuk, R., Morillon, E., et al. (2003b). LMO2-associated clonal T cell proliferation in two patients after gene therapy for SCID-X1. Science *302*, 415–419.

Harding, J., Roberts, R.M., and Mirochnitchenko, O. (2013). Large animal models for stem cell therapy. Stem Cell Res. Ther. *4*, 23.

Hiroyama, T., Miharada, K., Aoki, N., Fujioka, T., Sudo, K., Danjo, I., Nagasawa, T., and Nakamura, Y. (2006). Long-lasting in vitro hematopoiesis derived from primate embryonic stem cells. Exp. Hematol. *34*, 760–769.

Kaufman, D.S., Lewis, R.L., Hanson, E.T., Auerbach, R., Plendl, J., and Thomson, J.A. (2004). Functional endothelial cells derived from rhesus monkey embryonic stem cells. Blood *103*, 1325–1332.

Kennedy, M., Awong, G., Sturgeon, C.M., Ditadi, A., Lamotte-Mohs, R., Zuniga-Pflucker, J.C., and Keller, G. (2012). T lymphocyte potential marks the emergence of definitive hematopoietic progenitors in human pluripotent stem cell differentiation cultures. Cell Rep. *2*, 1722–1735.

Mavilio, D., Benjamin, J., Kim, D., Lombardo, G., Daucher, M., Kinter, A., Nies-Kraske, E., Marcenaro, E., Moretta, A., and Fauci, A.S. (2005). Identification of NKG2A and NKp80 as specific natural killer cell markers in rhesus and pigtailed monkeys. Blood *106*, 1718–1725.

Mendjan, S., Mascetti, V.L., Ortmann, D., Ortiz, M., Karjosukarso, D.W., Ng, Y., Moreau, T., and Pedersen, R.A. (2014). NANOG and CDX2 pattern distinct subtypes of human mesoderm during exit from pluripotency. Cell Stem Cell *15*, 310–325.

Mestas, J., and Hughes, C.C. (2004). Of mice and not men: differences between mouse and human immunology. J. Immunol. *172*, 2731–2738.

Natarajan, K., Dimasi, N., Wang, J., Mariuzza, R.A., and Margulies, D.H. (2002). Structure and function of natural killer cell receptors: multiple molecular solutions to self, nonself discrimination. Annu. Rev. Immunol. *20*, 853–885.

Nostro, M.C., Cheng, X., Keller, G.M., and Gadue, P. (2008). Wnt, activin, and BMP signaling regulate distinct stages in the developmental pathway from embryonic stem cells to blood. Cell Stem Cell *2*, 60–71.

Parham, P., Abi-Rached, L., Matevosyan, L., Moesta, A.K., Norman, P.J., Older Aguilar, A.M., and Guethlein, L.A. (2010). Primate-specific regulation of natural killer cells. J. Med. Primatol. *39*, 194–212.

Polychronopoulos, P., Magiatis, P., Skaltsounis, A.L., Myrianthopoulos, V., Mikros, E., Tarricone, A., Musacchio, A., Roe, S.M., Pearl, L., Leost, M., et al. (2004). Structural basis for the synthesis of indirubins as potent and selective inhibitors of glycogen synthase kinase-3 and cyclin-dependent kinases. J. Med. Chem. *47*, 935–946.

Rajesh, D., Chinnasamy, N., Mitalipov, S.M., Wolf, D.P., Slukvin, I., Thomson, J.A., and Shaaban, A.F. (2007). Differential requirements for hematopoietic commitment between human and rhesus embryonic stem cells. Stem Cells *25*, 490–499.

Seggewiss, R., Pittaluga, S., Adler, R.L., Guenaga, F.J., Ferguson, C., Pilz, I.H., Ryu, B., Sorrentino, B.P., Young, W.S., 3rd, Donahue, R.E., et al. (2006). Acute myeloid leukemia is associated with retroviral gene transfer to hematopoietic progenitor cells in a rhesus macaque. Blood *107*, 3865–3867.

Sharpe, M.E., Morton, D., and Rossi, A. (2012). Nonclinical safety strategies for stem cell therapies. Toxicol. Appl. Pharmacol. *262*, 223–231.

Shinoda, G., Umeda, K., Heike, T., Arai, M., Niwa, A., Ma, F., Suemori, H., Luo, H.Y., Chui, D.H., Torii, R., et al. (2007). alpha4-Integrin(+) endothelium derived from primate embryonic stem cells generates primitive and definitive hematopoietic cells. Blood *109*, 2406–2415.

Sturgeon, C.M., Ditadi, A., Awong, G., Kennedy, M., and Keller, G. (2014). Wnt signaling controls the specification of definitive and primitive hematopoiesis from human pluripotent stem cells. Nat. Biotechnol. *32*, 554–561.

Sumi, T., Tsuneyoshi, N., Nakatsuji, N., and Suemori, H. (2008). Defining early lineage specification of human embryonic stem cells by the orchestrated balance of canonical Wnt/beta-catenin, Activin/Nodal and BMP signaling. Development *135*, 2969–2979.

Timmermans, F., Velghe, I., Vanwalleghem, L., De Smedt, M., Van Coppernolle, S., Taghon, T., Moore, H.D., Leclercq, G., Langerak, A.W., Kerre, T., et al. (2009). Generation of T cells from human embryonic stem cell-derived hematopoietic zones. J. Immunol. *182*, 6879–6888.

Trobridge, G.D., and Kiem, H.P. (2010). Large animal models of hematopoietic stem cell gene therapy. Gene Ther. *17*, 939–948.

Uenishi, G., Theisen, D., Lee, J.H., Kumar, A., Raymond, M., Vodyanik, M., Swanson, S., Stewart, R., Thomson, J., and Slukvin, I. (2014). Tenascin C promotes hematoendothelial development and T lymphoid commitment from human pluripotent stem cells in chemically defined conditions. Stem Cell Rep. *3*, 1073–1084.

Umeda, K., Heike, T., Yoshimoto, M., Shiota, M., Suemori, H., Luo, H.Y., Chui, D.H., Torii, R., Shibuya, M., Nakatsuji, N., et al. (2004). Development of primitive and definitive hematopoiesis from nonhuman primate embryonic stem cells in vitro. Development *131*, 1869–1879.

Umeda, K., Heike, T., Yoshimoto, M., Shinoda, G., Shiota, M., Suemori, H., Luo, H.Y., Chui, D.H., Torii, R., Shibuya, M., et al. (2006). Identification and characterization of hemoangiogenic progenitors during cynomolgus monkey embryonic stem cell differentiation. Stem Cells *24*, 1348–1358.

Vodyanik, M.A., and Slukvin, I.I. (2007). Hematoendothelial differentiation of human embryonic stem cells. Curr. Protoc. Cell Biol. *Chapter 23*, Unit 23.26.

Vodyanik, M.A., Bork, J.A., Thomson, J.A., and Slukvin, I.I. (2005). Human embryonic stem cell-derived CD34+ cells: efficient production in the coculture with OP9 stromal cells and analysis of lymphohematopoietic potential. Blood *105*, 617–626.

Vodyanik, M.A., Thomson, J.A., and Slukvin, I.I. (2006). Leukosialin (CD43) defines hematopoietic progenitors in human embryonic stem cell differentiation cultures. Blood *108*, 2095–2105.

Vodyanik, M.A., Yu, J., Zhang, X., Tian, S., Stewart, R., Thomson, J.A., and Slukvin, I.I. (2010). A mesoderm-derived precursor for mesenchymal stem and endothelial cells. Cell Stem Cell *7*, 718–729.

Yu, J., Hu, K., Smuga-Otto, K., Tian, S., Stewart, R., Slukvin, I.I., and Thomson, J.A. (2009). Human induced pluripotent stem cells free of vector and transgene sequences. Science *324*, 797–801.

Yu, Q.C., Hirst, C.E., Costa, M., Ng, E.S., Schiesser, J.V., Gertow, K., Stanley, E.G., and Elefanty, A.G. (2012). APELIN promotes hematopoiesis from human embryonic stem cells. Blood *119*, 6243–6254.

Zhang, X.B., Beard, B.C., Trobridge, G.D., Wood, B.L., Sale, G.E., Sud, R., Humphries, R.K., and Kiem, H.P. (2008). High incidence of leukemia in large animals after stem cell gene therapy with a HOXB4-expressing retroviral vector. J. Clin. Invest. *118*, 1502–1510.

Stem Cell Reports, Volume *6*

Supplemental Information

GSK3β Inhibition Promotes Efficient Myeloid and Lymphoid Hematopoiesis from Non-human Primate-Induced Pluripotent Stem Cells

Saritha S. D'Souza, John Maufort, Akhilesh Kumar, Jiuchun Zhang, Kimberley Smuga-Otto, James A. Thomson, and Igor I. Slukvin

Supplementary Information

GSK3β Inhibition Promotes Efficient Myeloid and Lymphoid Hematopoiesis from Nonhuman Primate Induced Pluripotent Stem Cells

Saritha S. D'Souza, John Maufort, Akhilesh Kumar, Jiuchun Zhang, Kimberley Smuga-Otto, James A. Thomson, and Igor I. Slukvin

Supplementary Experimental Procedures

Embryonic Stem Cells.

Rh366.4 and Rh456 ESCs were derived from in vivo-flushed blastocysts (Thomson et al., 1995; Thomson and Marshall, 1998).

Immunofluorescence

iPSCs were washed with PBS, fixed in 1% paraformaldehyde at 4°C for 30 mins, and permeabilized in 90% methanol for 30 mins at -20°C. Cells were incubated with primary antibodies Oct 3/4, Sox2 and Nanog (see Supplementary Table S1) at 1:200 dilution in PBS with 2% FBS overnight at 4°C. Following washing with saline, cells were incubated with secondary antibodies (either donkey anti-rabbit alexa fluor 488 (Life Technologies) or donkey anti-mouse alexa fluor 568 (Life Technologies) antibodies at a 1:500 dilution in PBS with 2% FBS for 1 hour at room temperature. Images were captured using the EVOS FL Auto cell imaging system (Life Technologies). Alkaline Phosphatase staining was completed according to the manufacturers instructions, VECTOR Blue Alkaline Phosphatase (AP) Substrate Kit (VECTOR laboratories SK-5300).

Hematopoietic Differentiation of NHP iPSCs in defined conditions.

The iPSCs were differentiated in feeder free and chemically defined conditions as described previously (Uenishi et al., 2014). Briefly, single cell suspensions of iPSCs were plated at a density of 2,500 cells/cm^2 onto six well plates coated with a mixture of 0.25μg/cm^2 each of ColIV and TenC in Primate ES medium (Reprocell) supplemented with 4ng/ml FGF2 and 10μM Rho Kinase inhibitor (Tocris Y-27632). After 24hrs, the medium was changed to IF9S medium supplemented with 4μM CHIR99021, 15ng/ml Activin A, 50ng/ml FGF2, 2mM LiCl, 50ng/ml VEGF and 1μM Rho kinase inhibitor. The medium was then changed to IF9S medium supplemented with 50ng/ml of VEGF and 50ng/ml of FGF2 on day 2. IF9S medium supplemented with 50 ng/ml FGF2, VEGF, TPO, SCF, IL-6, and 10 ng/ml IL-3 was used on day 4. On day 6, additional IF9S medium supplemented with the same six factors were added to the cultures without aspirating the old medium. Differentiation was conducted in a hypoxic conditions from day 0 to day 4, and then in a normoxia in the remaining days. Cells were dissociated with 1x TrypLE and collected for analysis.

RNA Extraction and Quantitative RT-PCR

RNA was extracted with Illustra RNAspin mini RNA isolation kit (GE Healthcare). Equal amounts of RNA was used for cDNA synthesis using Quantitect Reverse Transcription kit (Qiagen) . The mRNA levels of the indicated genes were analyzed in triplicates using Power SYBR Green PCR master mix (Applied Biosystems). The reactions were run on a Mastercycler RealPlex Thermal Cycler (Eppendorf) and the expression levels were calculated by minimal cycle threshold values (Ct) normalized to the reference expression of β actin. The primer sequences are listed in supplementary Table S2.

Supplementary References

Thomson, J.A., Kalishman, J., Golos, T.G., Durning, M., Harris, C.P., Becker, R.A., and Hearn, J.P. (1995). Isolation of a primate embryonic stem cell line. Proc Natl Acad Sci U S A *92*, 7844-7848.

Thomson, J.A., and Marshall, V.S. (1998). Primate embryonic stem cells. Curr Top Dev Biol *38*, 133-165.

Uenishi, G., Theisen, D., Lee, J.H., Kumar, A., Raymond, M., Vodyanik, M., Swanson, S., Stewart, R., Thomson, J., and Slukvin, I. (2014). Tenascin C promotes hematoendothelial development and T lymphoid commitment from human pluripotent stem cells in chemically defined conditions. Stem cell reports *3*, 1073-1084.

Supplementary Figures:

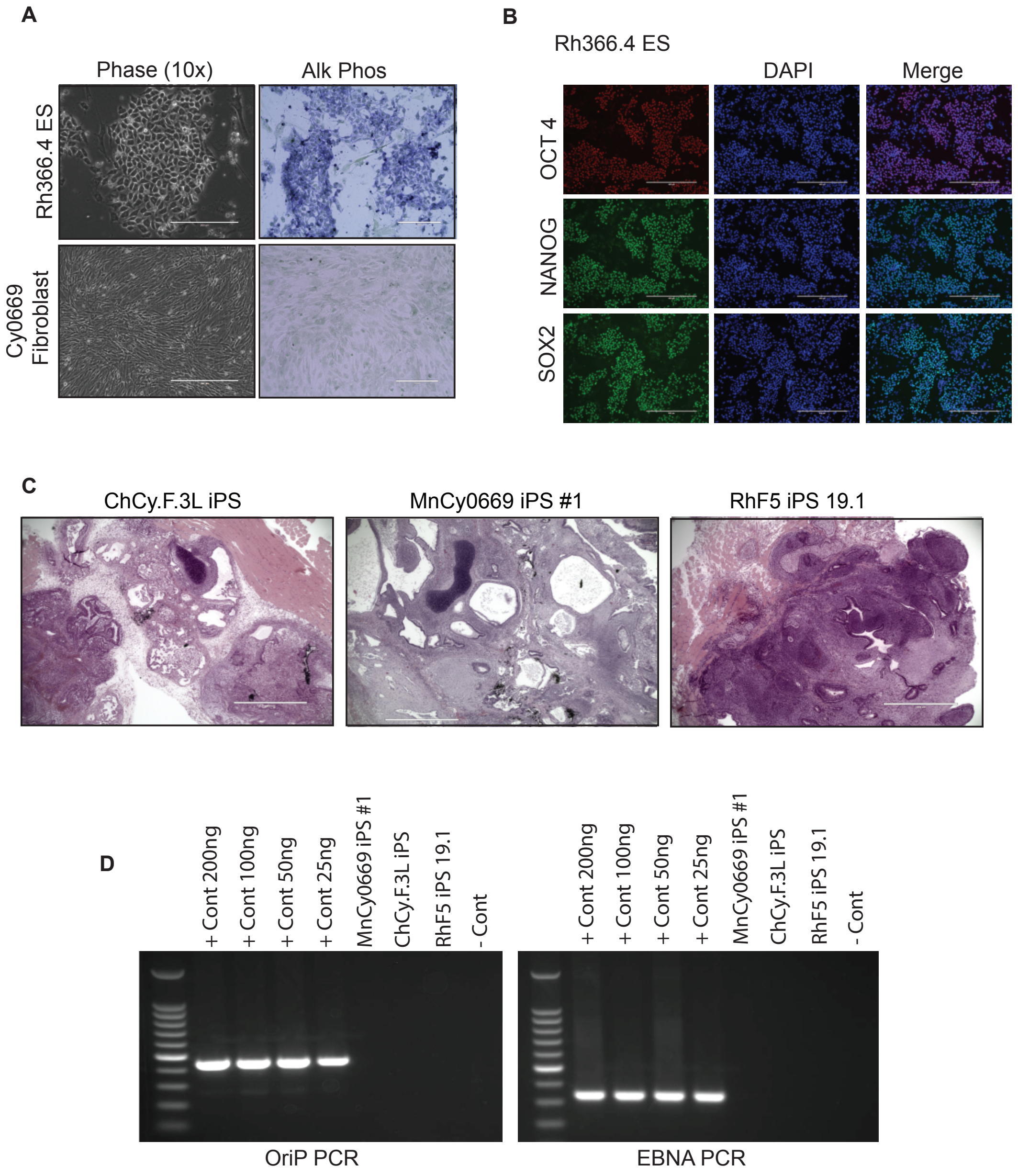

Supplementary Figure S1. Characterization of NHP iPSCs. (A) Morphology and alkaline phosphatase staining of rhesus ESCs. Fibroblasts from cynomolgus monkey served as a negative control. B) ES colonies from Rh366.4 were stained for pluripotency markers. (C) Low magnification images show teratoma formation by the indicated NHP iPSCs. (D) PCR analysis of iPSCs to confirm the absence of episomal reprogramming plasmids. Related to Figure 1.

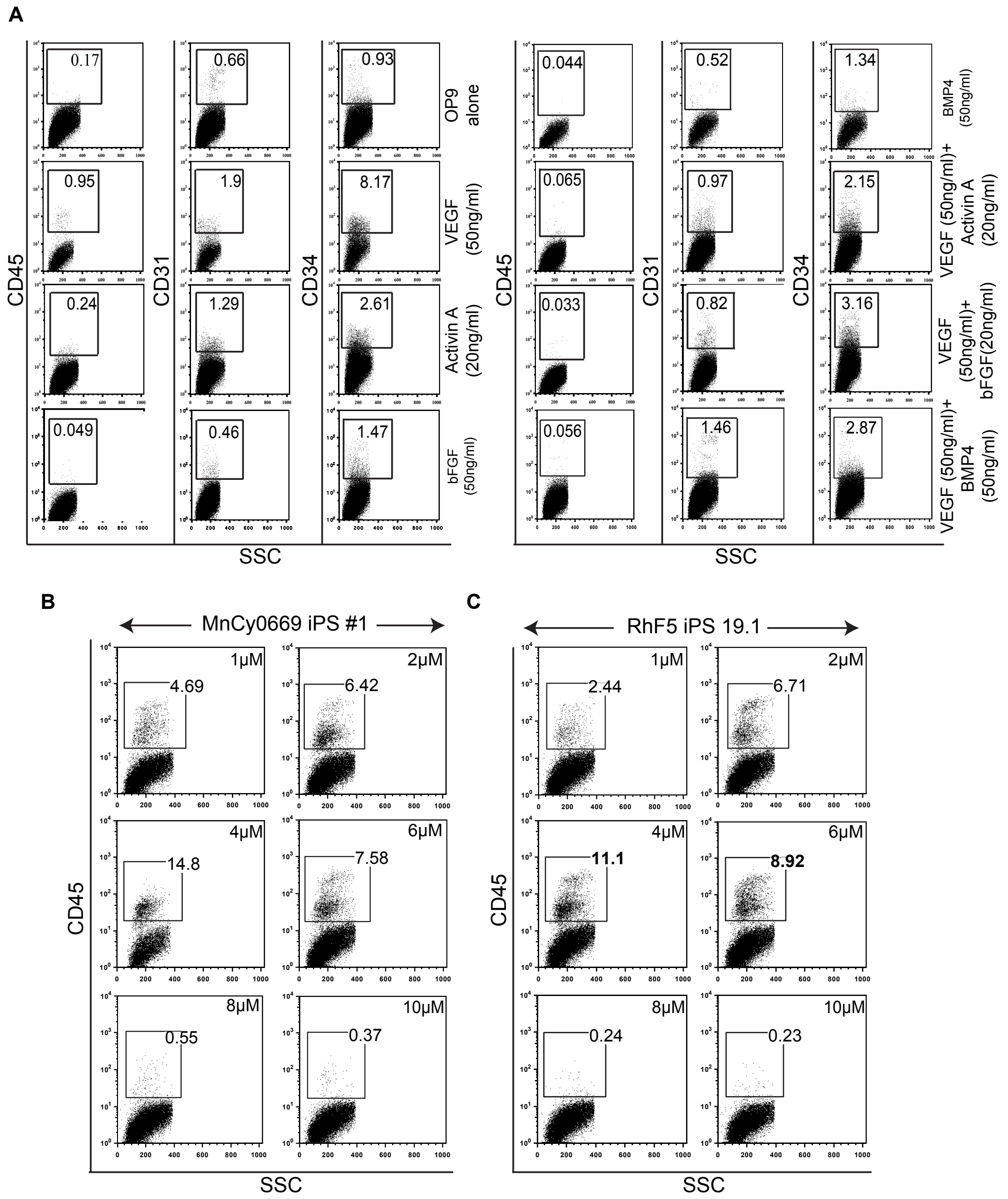

Supplementary Figure S2. Effect of various growth factors and CHIR99021 on hematopoiesis from NHP-iPSCs. (A) Comparative effect of different growth factor combinations on the generation of $CD45^+$ cells from MnCy0669 iPS#1 (B) Effect of different doses of CHIR99021 on hematopoietic differentiation of RhF5 iPS 19.1 and MnCy669 iPS#1. Related to Figure 2.

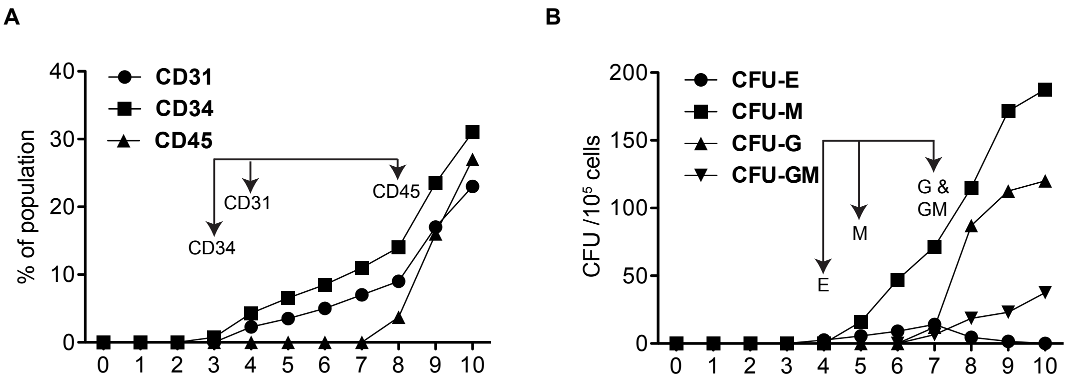

Supplementary Figure S3. (A) Percentages of the expression of CD45, CD34 and CD31 in Cy.F.3L iPSC/OP9 coculture following 10 days of differentiation as determined by flow cytometry. (B) Kinetics of different CFU types in Cy.F.3L iPSC/OP9 co-culture. Related to Figure 3.

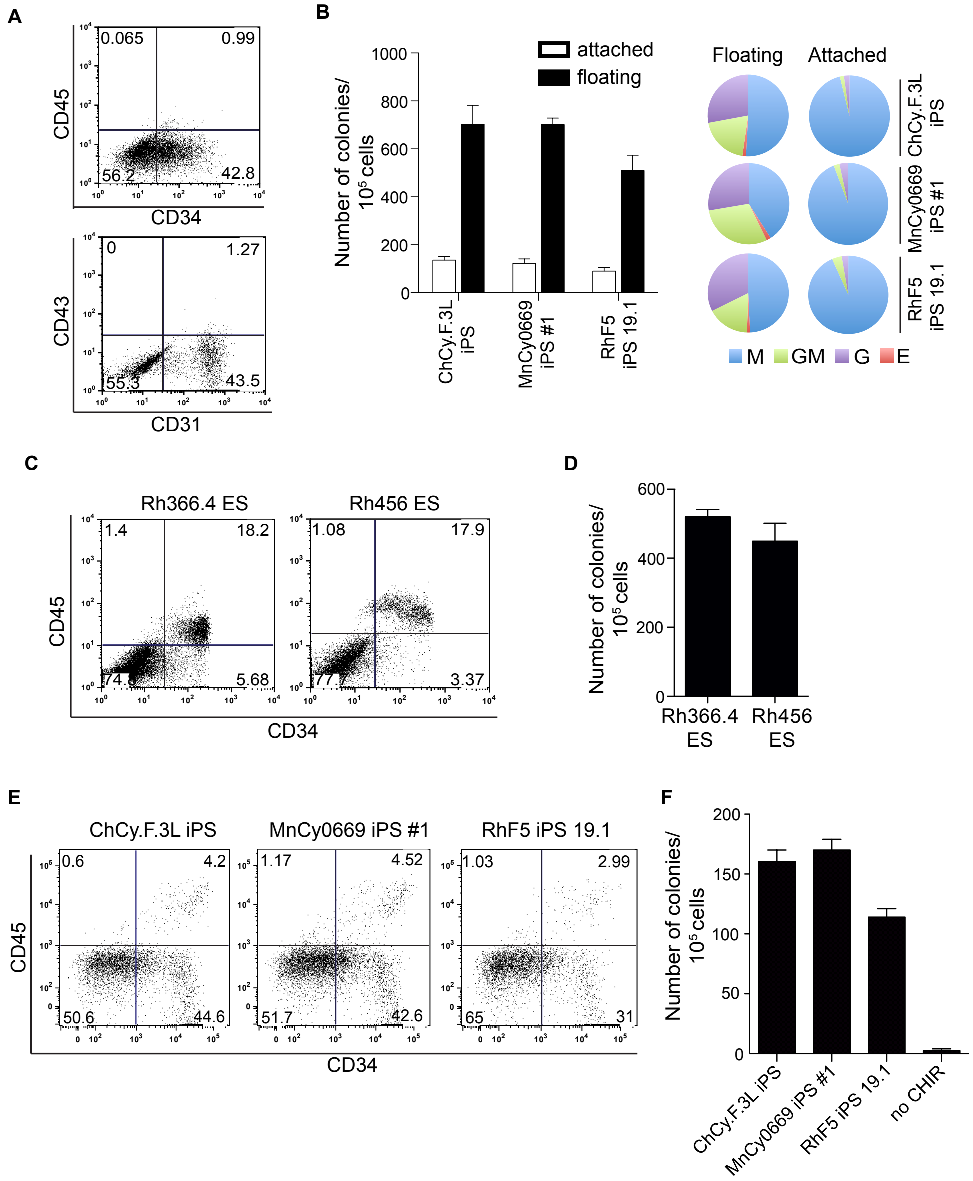

Supplementary Figure S4. Characterization of hematopoietic differentiation from NHP PSCs. (A) Flow cytometric analysis of the attached fraction after removal of floating cells on day 10 OP9-iPSC coculture. (B) CFU assay of the attached and floating fraction on day 10 of differentiation. Pie charts depict the relative proportions of CFU. Error bars are mean± SE from 3 independent experiments. (C) Rhesus ESCs Rh366.4 ES and Rh456 ES were differentiated on OP9 with CHR99021 and VEGF for 10 days and analyzed by flow cytometry. (D) CFU potential of the day 10 Rhesus ESCs differentiated on OP9 in presence of CHIR99021. Error bars are mean± SE from 3 independent experiments. (E) NHP iPSCs were differentiated on tenascin C and collagen IV coated plates in chemically defined conditions in the presence of CHIR99021 and VEGF (Uenishi et al., 2014). The cells were analyzed on day 8 of differentiation by flow cytometry. Cells differentiated without CHIR99021 gave rise to less than 0.5% of $CD45^+$ cells. (F) CFU analysis of NHP iPSCs differentiated in chemically defined conditions with CHIR99021. Typically, CFUs were not detected without CHIR99021, except few macrophage colonies in MnCy0669 iPS#1 (no CHIR bar). Error bars are mean± SE from 3 independent experiments. Related to Figure 3.

A

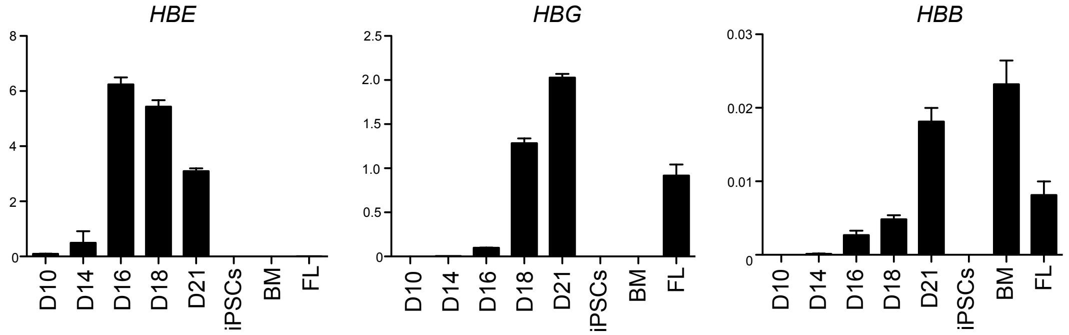

B

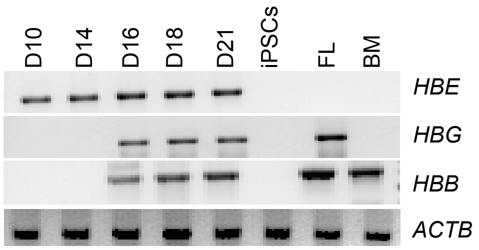

Supplementary Figure S5. Expression of embryonic ε globin (*HBE*), fetal γ globin (*HBG*) and adult β globin (*HBB*) in erythroid cultures from iPSCs. (A) qRT-PCR analysis of hemoglobins in cells cultures during erythroid differentiation. Relative expression normalized to β-actin (*ACTB*) is shown. Error bars are mean± SE from at least 3 experiments. BM (bone marrow) and FL (fetal liver) mononuclear cells were used as positive controls. (B) The PCR products were resolved on 1.5% agarose gel and visualized using ethidium bromide. Related to Figure 4.

Supplementary Table S1. List of Antibodies Used in Study, Related to Figures 1-5.

NAME	CLONE	COMPANY
Anti-NHP CD45	MB4-6D6<br>D058-1283	Miltenyi Biotech<br>BD- Biosciences
Anti-human CD34	563	BD- Biosciences
Anti-human CD-31	WM59	BD- Biosciences
Anti-human CD43	DFT-1	Acris Antibodies
Anti-human CD45RA	5H9	BD- Biosciences
Anti-human CD38	AT-1	StemCell Technologies
Anti-human CD90	5E10	BD- Biosciences
Anti-human APJ	72133	R&D Systems
Anti-human CD11b	ICRF44	BD- Biosciences
Anti-human CD71	L01.1	BD- Biosciences
Anti-human CD41a	HIP8	BD- Biosciences
Anti-human CD42a	ALMA.16	BD- Biosciences
Anti-human CD3ε	SP34	BD- Biosciences
Anti-human CD4	L200	BD- Biosciences
Anti-human CD5	UCHT2	Biolegend
Anti-human CD7	MT701	BD- Biosciences
Anti-rat TCRα/β	R73	Biolegend
Anti-human CD8	SK1	Biolegend
Anti-human CD56	B159	BD- Biosciences
Anti-human CD16	3G8	BD- Biosciences
Anti-human CD159a	Z199	Bekman Coulter
Anti-human Oct3/4	C10	Santa Cruz
Anti-human Nanog	D73G4	Cell Signaling
Anti-human Sox2	D6D9	Cell Signaling

Supplementary Table S2. Primer Sets Used in Study, Related to Figures 2 and 5 and Supplementary Figure S1

GENE	Accession Numbers	PRIMER SEQUENCE
KDR	XM_005555271.1	F: ATGCACGGCATCTGGGAATC<br>R: GTCACTGTCCTGCAAGTTGCTGTC
T	XM_001101421.2	F: GACAATTGGTCCAGCCTTG<br>R: GGGTACTGACTGGAGCTGGT
HBE	M81364.1	F:TGCATTTTACTGCTGAGGAGA<br>R:AAGAGAACTCAGTGGTACTT
HBG	M19433.1	F:CAGTTCCACACACTCGCTTCTGG<br>R:GTGATCTCTTAGCAGAATAGA
HBB	NM_001283367.1	F: ACACTTGCTTCTGACACAACTGT<br>R: ATTAGGCAGAATCCAGATCCTCA
RAG1	NM_000448.2	F: CCTGCTGAGCAAGGTACCTCA<br>R: ATCTGGGGCAGAACTGAGTCC
RAG2	XM_005578160.1	F: ACCTGGTTTAGCGGCAAAGA<br>R: TTTTGGGCCAGCCTTTTTGG
CD3E	AB583147.1	F: ACCTGTTCCCAACCCAGACT<br>R: GATCCTGCTGGCCTTTCCG
PRF1	XM_001107967.2	F: GAGGGGAGAGCACAAAGGAC<br>R: CGGATGTCCTCTCTTCACCG
IFNG	NM_001287657.1	F: TGACTCGAATGTCCAACGCA<br>R: CCCTATTTTAGCTGCTGGCG
EBNA	Custom designed	F: GAGGAACTGCCCTTGCTATT<br>R: CATCTCCATCACCTCCTTCATC
OriP	Custom designed	F: AGGCTACACCAACGTCAATC<br>R: GAGCACCTCACATACACCTTAC
ACTB	AY497558.1	F: GCAGGAGATGGCCACGGCGCC<br>R: TCTCCTTCTGCATCCTGTCGGC
